# Unilateral or bilateral drainage for patients with bilateral chronic subdural hematoma: a systematic review and retrospective cohort study

**DOI:** 10.1007/s10143-025-03530-0

**Published:** 2025-05-06

**Authors:** Merijn Foppen, K. Yah, K. M. Slot, P. van Schie, D. Verbaan, W. P. Vandertop

**Affiliations:** 1 Department of Neurosurgery, Amsterdam Neuroscience, Amsterdam University Medical Center, University of Amsterdam, Meibergdreef 9, Amsterdam, Netherlands; 2 Amsterdam Neuroscience, Neurovascular Disorders, Amsterdam, The Netherlands; 3 Amsterdam UMC, Location AMC, Meibergdreef 9, Room H2-241, Amsterdam, 1105 AZ The Netherlands

**Keywords:** Humans, Subdural, Hematoma, Chronic, Retrospective studies, Drainage, Reoperation, Systematic review

## Abstract

Bilateral chronic subdural hematoma (cSDH) can be treated with unilateral or bilateral drainage. Unilateral drainage reduces surgery-related risks but could entail growth of the contralateral, non-operated hematoma. This study aims to (1) determine the incidence of additional contralateral surgery, (2) find factors associated with its occurrence, and (3) evaluate outcomes of uni- and bilateral drainage in bilateral cSDH patients. A systematic review (SR) conducted according to the PRISMA guidelines, pooled incidences of additional contralateral surgery with 95% confidence intervals (CI). Meta-analyses explored factors associated with contralateral surgery. Subsequently, a single-center, retrospective cohort study of bilateral cSDH patients treated with burr hole craniostomy (2010–2022) was performed. The primary outcome was occurrence of additional, contralateral surgery after unilateral drainage. Additionally, surgical complications, 30-day mortality, and reoperation rates (ipsilateral recurrence and additional contralateral surgery) were compared for unilateral and bilateral approaches in all patients and in two subgroups: cases with clinical equipoise regarding surgical approach and a propensity score-matched cohort. The pooled incidence of additional contralateral surgery in 697 patients (SR: 630, cohort: 67) receiving unilateral surgery was 14% (95% CI: 9–19%). Smaller ipsilateral hematoma (mean difference (MD): 12.2 ml, 95% CI: 7.18–17.23) and larger contralateral hematoma (MD: -25.4 ml, 95% CI: -43.95/-6.85) were independently associated with contralateral treatment (meta-analysis SR). Among 178 patients of the retrospective cohort, bilateral drainage had higher complication rates (9.9% vs. 1.5%, *p* = 0.032). In 93 patients with clinical equipoise and in 56 propensity score-matched patients, surgical approach did not affect reoperation, complications, or 30-day mortality rate. In patients with bilateral cSDH, additional contralateral treatment is required in 14% of all patients. Bilateral surgery carried higher complication risks in our cohort, but in the two subgroups, unilateral or bilateral approaches yielded similar outcomes. Prospective studies are required to further define in which patients unilateral surgery may suffice.

**Supplementary Information:**

The online version contains supplementary material available at 10.1007/s10143-025-03530-0.

## Introduction

Bilateral chronic subdural hematoma (cSDH) accounts for approximately 16 to 35% of all cSDH cases [1–5]. When surgery is indicated, the neurosurgeon must decide whether to perform unilateral or bilateral evacuation, usually based on clinical and radiological features, such as laterality of symptoms, extent of midline shift and hematoma diameter and volume [5, 6]. Consensus within existing literature regarding the preference for unilateral or bilateral evacuation remains elusive.

The approach for treating bilateral cSDH lacks a universally accepted gold standard, although in Denmark a national guideline for managing cSDH has been developed, which advocates primary bilateral surgery for bilateral cSDH [7]. Several studies, including the aforementioned national guideline, suggest that opting for bilateral surgery significantly reduces the risk of growth and aggravation of the non-operated contralateral hematoma, which in turn could result in additional treatment [6, 7]. This proposition is based on the assumption that the contralateral hematoma might expand if only the larger ipsilateral hematoma is evacuated, leading to a shift in intracranial pressure and loss of contralateral counterbalance [8, 9]. Therefore, bilateral surgery is posited as a safeguard against eventual contralateral treatment in bilateral cSDH patients. However, bilateral evacuation might also increase the risk of surgical complications, such as a postoperative acute subdural hematoma (aSDH), extended surgery time, longer hospital stays and increased likelihood of wound infection or leakage [10–12].

The main objective of this study is to determine the incidence of additional contralateral treatment after initial unilateral surgery in patients with bilateral cSDH and to evaluate the effect of surgical approach (initial unilateral or bilateral surgery) on clinical outcome. To do this, we systematically reviewed the existing literature and, in addition, evaluated the data of our own retrospective cohort.

## Methods

### Systematic review: search

First, a systematic review of the literature concerning contralateral treatment after initial unilateral surgery in patients with a bilateral cSDH, was conducted according to the PRISMA guidelines [13]. The protocol of the systematic review was registered to the PROSPERO-database. The search for relevant studies was carried out using different search engines (MEDLINE, Embase Cochrane Library, Scopus and CINAHL). Literature between database inception and May 30th, 2024 was searched (see Supplement A for the complete search strategy). Studies were included if (1) they contained patients with a bilateral cSDH, (2) patients were older than 18 years, (3) initial treatment was unilateral surgical drainage with BHC, craniotomy or twist-drill craniostomy. Studies were excluded if (1) they contained < 10 patients, (2) they contained patients treated with tranexamic acid, dexamethasone or middle meningeal artery embolization, (3) the language was other than English or Dutch, (4) they contained patients who received an epidural block for delivery or any other indications, (5) the contralateral treatment rate was not reported, (6) the full-text was not available, (7) certain publication types (letter to the editor, commentary, survey, study protocol) were involved. Two investigators (M.F. and K.Y.) independently screened title and abstract to identify potential eligible studies. Records lacking abstracts were automatically directed to the full-text screening phase. Subsequently, full texts were independently screened by two investigators (M.F. and K.Y.) based on the inclusion and exclusion criteria. Any disagreements between the two investigators were sorted by discussion. A third assessor (D.V.) was consulted in case of any discrepancies.

### Systematic review: data collection and outcomes

The following data was extracted from the included studies: study information (authors, year of publication, study design), patient characteristics (age, sex, use of anticoagulant or antiplatelet therapy, GCS), radiological parameters (midline shift, hematoma diameter and volume), surgical details (type of surgery and drain use), and number of patients receiving contralateral treatment and time until contralateral treatment. The systematic review determined two outcomes: the incidence of additional contralateral surgery in patients who received initial unilateral surgery, which is the primary outcome of this study, and factors associated herewith. Contralateral treatment was defined as: ‘the need for surgical treatment of the contralateral cSDH, including bilateral surgery, during follow-up’. For both outcomes, data of our own cohort study were also included in the results of the systematic review.

### Systematic review: quality assessment

Risk of bias and quality assessment were assessed by scoring each study according to the Newcastle-Ottawa scale (NOS), which is validated for assessing the quality of observational cohort studies. Then, the NOS was converted to Agency for Healthcare Research and Quality (AHRQ-) standards [14]. The classification was as follows: good quality indicated 3 or 4 stars in selection domain, 1 or 2 stars in comparability domain, and 2 or 3 stars in outcome/exposure domain. Fair quality indicated 2 stars in selection domain, 1 or 2 stars in comparability domain, and 2 or 3 stars in outcome/exposure domain. For poor quality, the rankings were 0 or 1 stars, 0 stars, or 0 or 1 stars in each domain, respectively. Bilateral evacuated cSDH patients were determined to be the non-exposed cohort. In case there was no non-exposed cohort in an included study, the maximum star allocation was capped at 8. Consequently, the subsequent categorization for these studies was 2 or 3 stars for good quality, 1 or 2 stars for fair quality, and 0 stars for poor quality in the selection domain. The star allocation in the other domains remained the same. The data extraction and quality assessment were conducted independently by two investigators (M.F. and K.Y.). Any discrepancies were sorted by discussion.

### Cohort study: patient selection

**Fig. 1:**
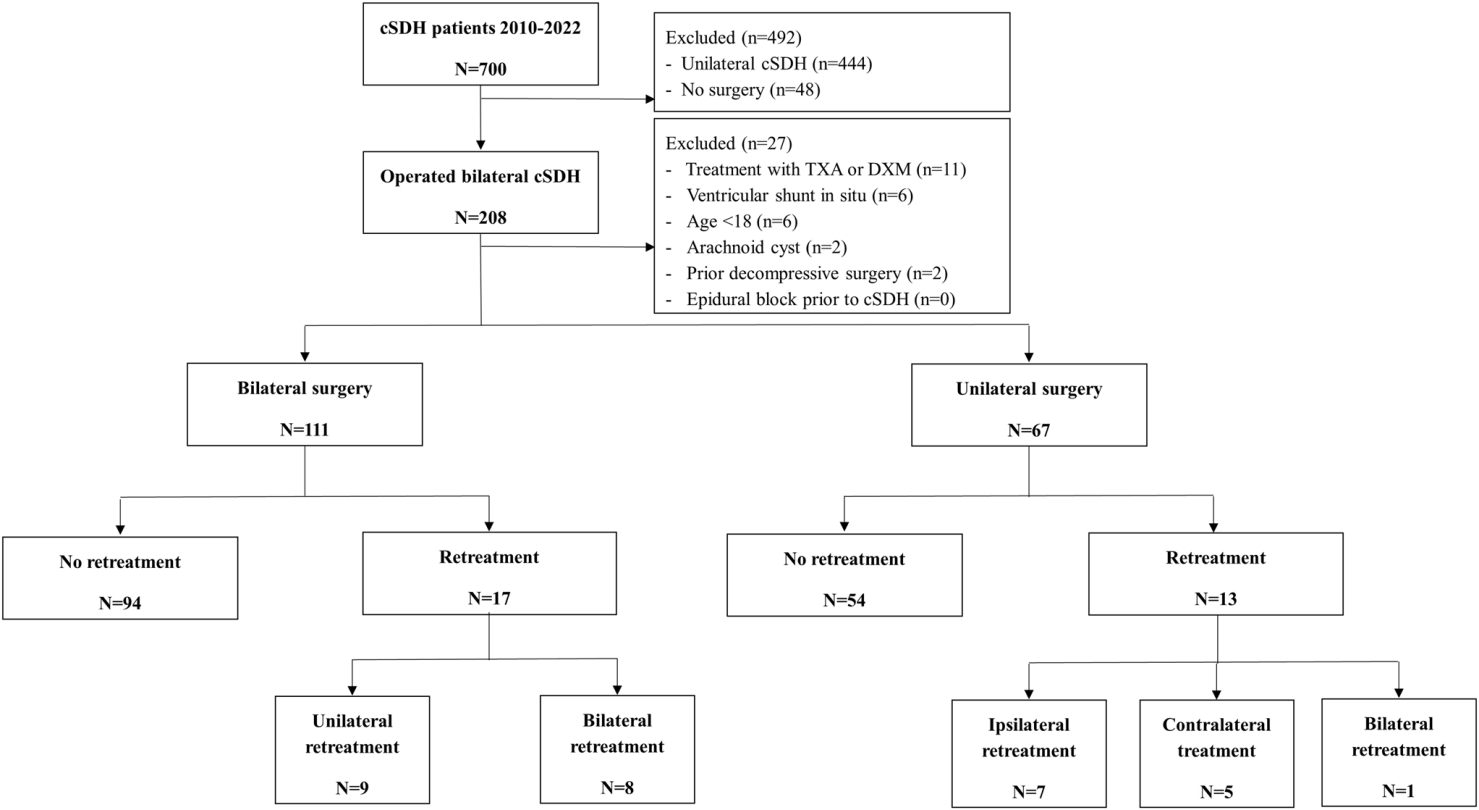
Flowchart of patient selection

Additionally, a single-center, cohort study was performed in Amsterdam University Medical Center (AUMC), the largest academic hospital in the Netherlands. Patients with a bilateral cSDH who received surgical treatment (burr hole craniostomy (BHC) were selected from a retrospective database. This database contains all cSDH patients treated at this center between 2010 and 2022. Patients were excluded if they (1) were less than 18 years old, (2) received off-label pharmacological treatment with tranexamic acid or dexamethasone, (3) had a cerebrospinal fluid shunt in situ, (4) had received cranial decompressive surgery or surgery for a primary cSDH in the year prior to diagnosis (5) had an arachnoid cyst or other structural abnormality, (6) had an epidural block prior to their cSDH, or (7) were included in ongoing randomized controlled trials (TORCH- and ELIMINATE-study [15, 16]) (Fig. 1). Formal approval for this study was waived off by the local ethics committee and a waiver for informed consent was obtained (waiver number 2023.1006, clinical trial number: not applicable).

### Cohort study: surgical procedure

All patients were treated with single or double burr hole craniostomy with post-operative drainage. Pre-operatively, patients were administered two grams of intravenous Cefazolin and, if required, antiplatelet and/or anticoagulant therapy was stopped and reversed. The surgery was performed under general anesthesia. Saline was used as intra-operative irrigation fluid, and a subdural or subgaleal drain was placed for post-operative drainage. It was standard practice to remove the drain after 24 h. The decision to perform uni- or bilateral surgery was made by the attending neurosurgeon, and based on laterality and severity of symptoms, in combination with the radiological appearance of the hematomas. No institutional algorithms or pre-specified criteria were used in this decision. For bilateral surgery, it was at the discretion of the neurosurgeon to decide whether to perform both sides concurrently, or in a staged manner with separate patient repositioning.

### Cohort study: outcomes

In the cohort study, the results per surgical approach, initial unilateral or bilateral surgery, were evaluated. Outcomes included surgical complications (post-operative aSDH diagnosed by Computed Tomography (CT), wound infection (if antibiotic treatment was started or if wound cultures were positive) and leakage), 30-day mortality and reoperation rate. General complications such as pneumonia and other infections, and electrolyte disturbances were not included. For patients who received initial bilateral surgery, reoperation rate was defined as: ‘additional surgery for a recurrent cSDH on either side during follow-up.’ For patients who received initial unilateral surgery, reoperation rate was defined as: ‘additional surgery for cSDH due to ipsilateral recurrence, or additional surgery due to clinical or radiological progression of the hematoma on the contralateral side, during follow-up’. If reoperation was performed bilaterally, this was considered as one surgery.

### Cohort study: data collection

The medical records of all included patients were examined, collecting demographic information (age, sex), medical history (cardiac arrhythmia, cerebral vascular accident (CVA), ischemic heart disease, venous thromboembolism (VTE), chronic obstructive pulmonary disease (COPD), diabetes, hypertension, malignancy, alcoholism in history, usage of anticoagulant or antiplatelet therapy, clinical features at diagnosis (Glasgow Coma Scale (GCS)), presence of headache, Markwalder Grading Scale (MGS), motor and sensory deficits and pre-operative radiological features (hematoma laterality, presence and amount of midline shift, hematoma diameter and volume). Hematoma volume was measured using Brainlab software (Brainlab AG, Munich, Germany). The volume relation (VR) ratio was calculated by dividing the volume of the smaller hematoma by the volume of the larger, ipsilateral hematoma (based on the method of a previous study by Scheichel et al. [5]). The advantage of this ratio is that it diminishes dependence on individual patient factors such as brain atrophy and elasticity, and that it demonstrates the relative relationship between both hematomas. Reoperation rate, additional contralateral treatment, length of follow-up (from date of diagnosis until last neurological or neurosurgical outpatient follow-up visit, or last day of clinical admission or presentation), surgical complication rate and 30-day mortality were also collected from the patients’ medical file. Data was pseudonymized and stored online using Castor EDC, an online case report form system [17].

### Statistical analysis and meta-analyses

In the literature analysis, the pooled incidence of contralateral treatment was determined by performing a single-arm meta-analysis and by creating a forest plot with 95% confidence interval (CI). To assess potential publication bias a funnel plot was created by plotting the contralateral surgery rate against the standard error. To explore factors associated with contralateral treatment, additional meta-analyses were conducted. To be included in the meta-analyses, variables had to be reported in at least two studies. For continuous variables mean differences (MD) with 95% CIs were calculated, and odds ratios (OR) with 95% CI for dichotomous variables. For all meta-analyses a random-effects model was used and heterogeneity was assessed using the *χ*^*2*^ test and *I*^*2*^-test. For the *χ*^*2*^ test, heterogeneity was considered significant if *p* < 0.05. For the *I*^*2*^-test, heterogeneity was considered low if *I*^*2*^ was 0–25%, mild for 25–50% and high > 50% [18]. In the retrospective cohort, normality of continuous data was assessed using the Shapiro-Wilk test, and data was considered normally distributed with a test value > 0.9. For continuous outcomes a mean and standard deviation (SD) were calculated for normally distributed data. For not-normally distributed data a median and interquartile range (IQR) were calculated. Baseline characteristics between groups (initial unilateral drainage vs. initial bilateral drainage and contralateral treatment vs. no contralateral treatment) were assessed using appropriate tests (independent samples t-test, Mann-Whitney U test, Chi-squared test and Fisher’s exact test). Subsequently, two subgroups were established. In some patients with bilateral cSDH, surgical approach is evident, but in others it remains uncertain (clinical equipoise, first subgroup). To identify the patients to whom this applies, all cases of the retrospective study were presented to three neurosurgeons (MSL, PVS, WVA, supplement B, Fig. 1), who were blinded to surgical approach and outcomes. Per case, the neurosurgeons individually determined whether they would perform unilateral or bilateral surgery, or whether both approaches were possible. If their responses were conflicting, or if all three indicated that both a uni- or bilateral approach were considered a valid treatment option (supplement B, Table 1), this was considered clinical equipoise. In addition, propensity score matching (PSM) was performed to establish the second subgroup. The covariates selected for the propensity score had to have a p-value of < 0.05 in univariate analyses of the total population (supplement B, Table 2). We did not include smallest hematoma diameter and VR-ratio, because both variables were already represented by volume of the smallest hematoma, which was also included in the propensity score as covariate. Matching was performed with the use of an optimal 2:1 matching protocol without replacement and a caliper width equal to 0.2. The primary outcomes were complications, 30-day mortality and reoperation rate and were compared using Fisher and Chi-squared tests in all patients and in the two subgroups (the clinical equipoise and propensity score-matched group). For the statistical analyses, the data were processed using SPSS (version 28.0, IBM, Armonk, New York, United States) and R studio version 4.2.1 (R Foundation for Statistical Computing, Vienna, Austria) and the ‘meta’ and ‘MatchThem’ package [19].

## Results

### Systematic review and meta-analysis

**Fig. 2:**
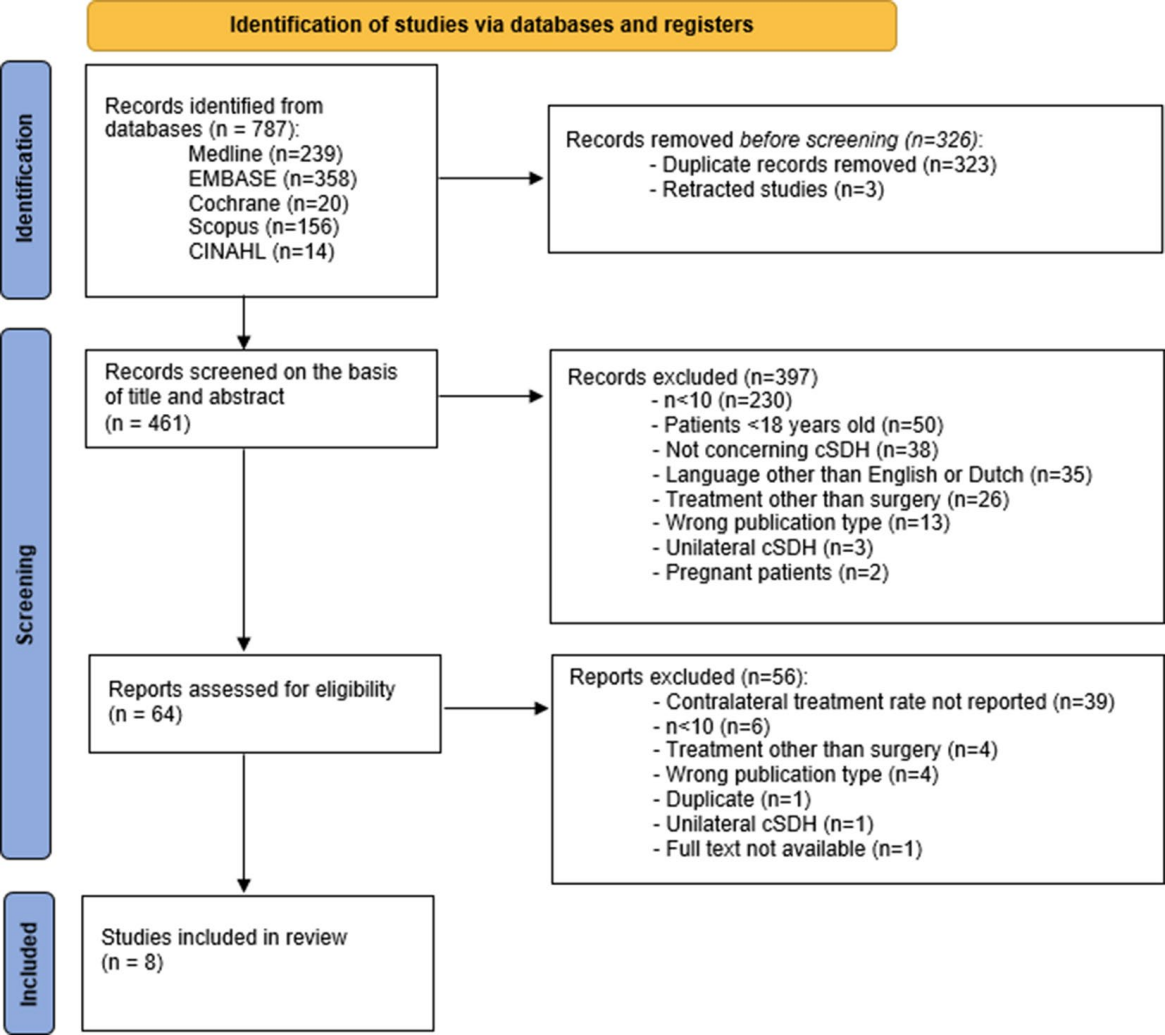
PRISMA flow diagram of study selection

A total of 787 studies were identified through database searching. Three hundred twenty-six studies were excluded, of which 232 were duplicates, and three studies were retracted. This resulted in the assessment of 461 studies based on title and abstract. Subsequently, 397 studies were excluded, leaving 64 studies for eligibility assessment. After full-text screening, eight studies were included (Fig. 2).

All included studies were retrospective cohort studies and contained a total of 630 patients (Table 1).

**Table 1:** Baseline characteristics and outcomes of eight included studies

Study	**Type**	**N**	**Contralateral treatment**	**Indication for unilateral surgery**	**Initial surgery type**	**Surgical drain**	**Follow-up in months**	**Time until contralateral treatment**	
Andersen-Ranberg et al., 2017 [6]	R	136	23	NA	BHC (94.6%) or craniotomy	Subgaleal or subdural, 24–48 h	12	34 days (median)	
Fujitani et al., 2017 [8]	R	93	18	If (1) the contralateral hematoma diameter was < 10 mm, (2) laterality of symptoms was due to the thicker hematoma, (3) the contralateral hematoma was restricted to the frontal side.	BHC	Silicone tube, 24 h	6	46 days (median)	
Langroudi et al., 2018 [3]	R	77	7	Hematoma diameter > 10 mm, uni- or bilateral symptoms.	BHC or craniotomy*	Yes, Jackson-Pratt/Blake Bard	2	25 days (range 3–63)	
Scheichel et al., 2018 [5]	R	42	9	NA	BHC (88.5%) or craniotomy	Yes, type not specified	6	28 days (SD 16.7)	
Takahashi et al., 2018 [20]	R	22	5	If laterality of symptoms could only be attributed to the thicker hematoma.	BHC	Yes, 24 h	6	38 days (SD 34.1)	
Shen et al., 2019 [21]	R	53	15	Size of the contralateral hematoma, symptoms and midline shift.	BHC	Yes, silicone tube (max 72 h)	14	NA	
Blaauw et al., 2020 [22]	R	109	12	NA	BHC (97%), craniotomy and TDC	Yes, subdural, 24–48 h	3	NA	
Zhang et al., 2020 [1]	R	98	4	NA	BHC (72.4%) and craniotomy	Yes, subgaleal or subdural	6	31 (range 24–35)	
Total		630	93						

*R: retrospective cohort study; NA = not applicable; BHC = burr hole craniostomy; TDC = twist drill craniostomy*, *percentage of patients treated with BHC not reported

**Fig. 3:**
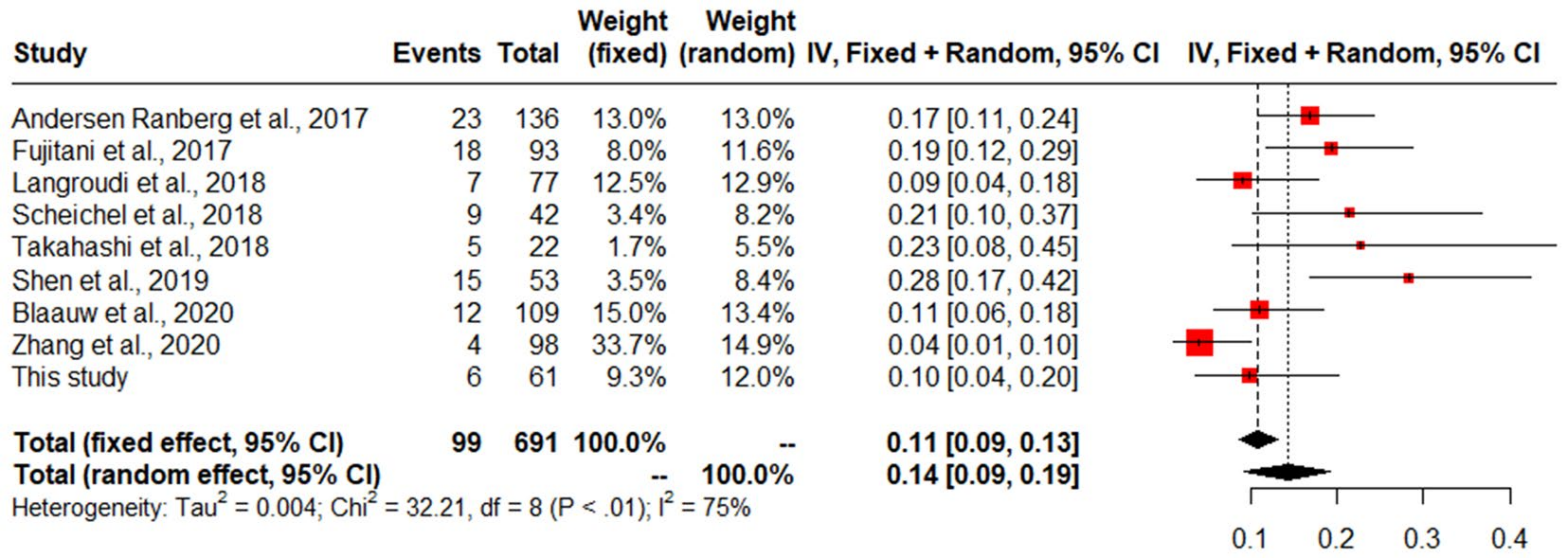
Pooled incidence of additional contralateral treatment after initial unilateral surgery. Apart from the eight studies found through the literature search, data from unilaterally treated patients of our own cohort was also added. These results are postulated under ‘this study’

The risk of bias scores are shown in Supplement C for the results of the quality assessment. Five included studies had a good quality and three a poor quality. The pooled incidence of contralateral treatment rate, including the data of our own cohort, was 14% (*n* = 697, 95% CI 9–19%) (Fig. 3).

**Fig. 4:**
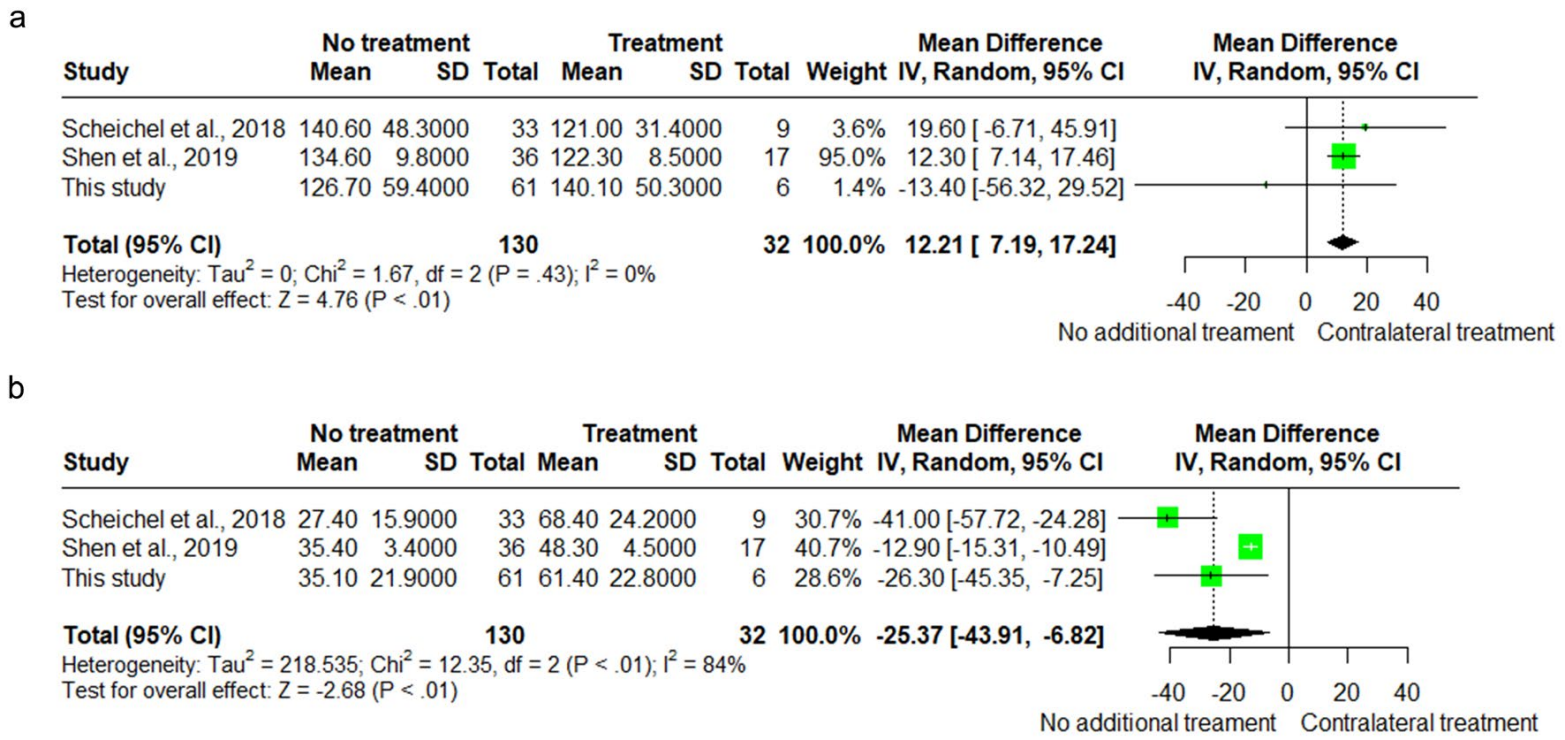
**a**. Forest plot ipsilateral hematoma volume. Data used from our own cohort is postulated under ‘this study’. **b**. Forest plot contralateral hematoma volume. Data used from our own cohort is postulated under ‘this study’

Subsequently, meta-analyses were conducted to investigate which parameters were associated with additional contralateral treatment, in bilateral cSDH patients treated with initial unilateral surgery. These factors comprised age, sex, usage of anticoagulant or antiplatelet therapy, midline shift, ipsilateral and contralateral hematoma diameter, as well as ipsilateral and contralateral hematoma volume. According to the meta-analysis findings, a smaller ipsilateral hematoma volume (MD 12.2 ml, 95% CI 7.18–17.23) and a larger contralateral hematoma volume (MD -25.4 ml, 95% CI -43.95/-6.85) were both independently associated with additional contralateral treatment (Fig. 4a and b). See supplement D for the meta-analyses of all other factors.

### Retrospective cohort

In our own cohort, a total of 178 (supplement B, Table 2) patients with bilateral cSDH were included, of whom 67 received initial unilateral surgery. The median length of follow-up was 63 days (IQR 47–91). In these patients, there was no significant difference in the reoperation rate (19.4% for unilateral surgery vs. 15.3% for bilateral surgery, *p* = 0.48) or the 30-day mortality rate (3.0% for unilateral surgery vs. 3.9% for bilateral surgery, *p* = 1.000). However, the complication rate was notably higher in the group that underwent initial bilateral surgery, compared to the group that had initial unilateral surgery (9.9% vs. 1.5%, *p* = 0.032). See supplement B, Table 3, for the outcomes in all patients of the cohort study. Six (9%) patients required additional contralateral treatment (supplement B, Table 4) and the median time between initial unilateral surgery and additional contralateral treatment was 32 days (IQR 12–51). See supplement B “clinical course”, for a detailed description of the clinical course of the six patients requiring additional contralateral surgery.

In 93 patients, there was clinical equipoise with regard to surgical approach (uni- or bilateral surgery). Eighteen patients (19%) received initial unilateral surgery and 75 bilateral surgery (Table 2).

**Table 2:** Baseline characteristics of 93 patients with presumed equipoise concerning surgical approach

	*Total (n = 93)*	*Bilateral surgery (n = 75)*	*Unilateral surgery (n = 18)*	*p*-value
Age (SD)	74.1 (10.8)	74.6 (10.2)	72.1 (13.1)	0.369^a^
Male (%)	71 (71.3)	68 (77.3)	13 (72.2)	0.881^b^
*History*				
Arrhythmia (%)	22 (22.6)	13 17.3)	8 (44.4)	0.025^d^
CVA (%)	17 (18.3)	14 (18.7)	3 (16.7)	1.000^d^
Ischemic heart disease (%)	17 (18.3)	14 (18.7)	3 (16.7)	1.000^d^
VTE/PE (%)	2 (2.2)	0 (0)	2 (11.1)	0.036^d^
COPD (%)	12 (12.9)	10 (13.3)	2 (11.1)	1.000^d^
Diabetes mellitus (%)	22 (23.7)	18 (24)	4 (22.2)	1.000^d^
Hypertension (%)	40 (43)	30 (40)	10 (55.6)	0.292^d^
Malignancy (%)	20 (21.5)	16 (21.3)	4 (22.2)	1.000^d^
Alcoholism in history (%)	7 (7.5)	6 (8)	1 (5.6)	1.000^d^
*Medication*				
Anticoagulant or antiplatelet therapy (%)	45 (48.4)	33 (44)	12 (66.7)	0.143^b^
*Clinical features at diagnosis*				
MGS (%)^89^				0.012^d^
1	34 (38.2)	28 (39.4)	6 (33.3)	
2	52 (58.3)	43 (60.6)	9 (50)	
3	3 (0.4)	0 (0)	3 (16.7)	
*Pre-operative radiological features*				
Midline shift (%)^92^	59 (64.1)	42 (56.8)	17 (94.4)	0.007^b^
Midline shift in mm (IQR)^59^	5 (3–6)	2 (0-4.5)	6 (5–8)	< 0.001^c^
Hematoma diameter largest hematoma in mm (SD)	19.1(7.8)	19.1 (7.4)	18.9 (9.6)	0.942^a^
Hematoma diameter smallest hematoma in mm (SD)	14.1 (5.4)	14.8 (5.3)	11.2 (5.2)	0.012^a^
Hematoma volume largest hematoma in ml (SD)^82^	123.5 (53.2)	120 (45.5)	139.4 (79.3)	0.203^a^
Hematoma volume smallest hematoma in ml (SD)^82^	74.9 (32.2)	80.8 (30.9)	48.7 (24.2)	< 0.001^a^
Volume relation ratio (SD) ^82^	0.65 (0.20)	0.70 (0.16)	0.43 (0.22)	< 0.001^a^

*With superscript in the column*,* ‘variable’ is indicated for how many patients data was available. CVA*,* cerebrovascular accident; VTE*,* ventous thrombolic embolism; PE*,* pulmonary embolism; COPD*,* chronic obstructive pulmonary disease; MGS*,* Markwalder Grading Scale.* ^*a*^ *Unpaired T-test*, ^*b*^*Chi-squared test*, ^c^*Mann-Whitney U-test*, ^*d*^ *Fisher’s exact test*

Patients receiving unilateral surgery had a significantly higher Markwalder Grading Scale and more midline shift (median 6 mm vs. 2 mm, *p* < 0.001). Also, the smallest hematoma was significantly smaller, with a volume of 48.7 ml versus 80.8 ml (*p* < 0.001). Upon comparison of initial unilateral and bilateral surgery, no significant differences were found in reoperation (16.7% vs. 10.7%), complication (5.6% vs. 13.3%), or 30-day mortality rates (5.6% vs. 2.7%) (Table 3).

**Table 3:** Outcomes according to surgical approach in the clinical equipoise group

*Outcome*	*Bilateral surgery (n = 75)*	*Unilateral surgery (n = 18)*	*p-value*
Reoperation (%)	8 (10.7)	3 (16.7)	0.440^*a*^
Complications (%)	10 (13.3)	1 (5.6)	0.684^*d*^
Post-operative aSDH (%)	3 (4)	1 (5.6)	1.000^*d*^
Post-operative wound infection or leakage (%)	4 (10)	0 (0)	0.085^*d*^
30-day mortality^9^ (%)	2 (2.7)	1 (5.6)	0.474^*d*^

*Complications are a composite of post-operative aSDH and wound infection or leakage. In 9 patients 30-day mortality could not be determined because of a shorter follow-up.* ^*a*^*Chi-squared test*, ^*d*^*Fisher-test*

The propensity score-matched cohort included 56 patients of whom 33 received bilateral surgery (Table 4). No significant baseline imbalances between the two groups were observed and there was no significant difference in reoperation, complication or 30-day mortality rate (Table 5).

**Table 4:** Baseline characteristics of 56 patients after propensity score matching

	*Total (n = 56)*	*Bilateral surgery (n = 33)*	*Unilateral surgery (n = 23)*	*p*-value
Age (SD)	72.3 (11.1)	71.1 (10.2)	74.1 (12.3)	0.330^a^
Male (%)	14 (25.0)	10 (30.3)	4 (17.4)	0.433^b^
*History*				
Arrhythmia (%)	16 (28.6)	7 (21.1)	9 (39.1)	0.229^c^
CVA (%)	14 (25.0)	8 (24.2)	6 (26.1)	1.000^c^
Ischemic heart disease (%)	14 (25.0)	8 (24.2)	6 (26.1)	1.000^c^
VTE/PE (%)	6 (10.7)	3 (9.1)	3 (13.0)	0.681^c^
COPD (%)	5 (8.9)	4 (12.1)	1 (4.3)	0.639^c^
Diabetes mellitus (%)	17 (30.4)	7 (21.2)	10 (43.5)	0.087^c^
Hypertension (%)	13 (23.2)	14 (42.4)	12 (52.2)	0.588^c^
Malignancy (%)	5 (8.9)	8 (24.2)	5 (21.7)	1.000^c^
Alcoholism in history (%)		4 (12.1)	1 (4.3)	0.639^c^
*Medication*				
Anticoagulant or antiplatelet therapy (%)	37 (66.1)	21 (63.6)	16 (69.6)	0.862^b^
*Clinical features at diagnosis*				
MGS (%)				0.078^d^
0	3 (5.4)	0 (0)	3 (13.0)	
1	18 (32.1)	10 (30.3)	8 (34.8)	
2	30 (53.6)	21 (63.6)	9 (39.1)	
3	5 (8.9)	2 (6.1)	3 (13.0)	
*Pre-operative radiological features*				
Midline shift in mm (IQR)	5 (2–7)	5 (2–6)	6 (2-7.5)	0.620^c^
Hematoma diameter largest hematoma in mm (SD)	18.1 (7.8)	18.5 (7.3)	17.5 (8.5)	0.641^a^
Hematoma diameter smallest hematoma in mm (SD)	12.3 (5.2)	12.8 (4.5)	11.5 (6.0)	0.371^a^
Hematoma volume largest hematoma in ml (SD)	119.3 (58.7)	114.0 (46.3)	127 (73.4)	0.418^a^
Hematoma volume smallest hematoma in ml (SD)	56.3 (19.2)	57.4 (17.4)	54.8 (21.8)	0.609^a^
Volume relation ratio (SD)	0.53 (0.20)	0.55 (0.20)	0.50 (0.21)	0.369^a^

*CVA*,* cerebrovascular accident; VTE*,* ventous thrombolic embolism; PE*,* pulmonary embolism; COPD*,* chronic obstructive pulmonary disease; MGS*,* Markwalder Grading Scale.* ^*a*^ *Unpaired T-test*, ^*b*^*Chi-squared test*, ^c^*Mann-Whitney U-test*, ^*d*^ *Fisher’s exact test*

**Table 5:** Outcomes in the propensity score-matched cohort

*Outcome*	*Bilateral surgery (n = 33)*	*Unilateral surgery (n = 23)*	*p-value*
Reoperation (%)	5 (15.1)	3 (13.0)	1.000^d^
Complications (%)	4 (12.1)	0 (0)	0.135^d^
Post-operative aSDH (%)	0 (0)	0 (0)	1.000^d^
Post-operative wound infection or leakage (%)	4 (0)	0 (0)	0.135^d^
30-day mortality^6^ (%)	1 (3.6)	1 (4.5)	1.000^d^

*Complications are a composite of post-operative aSDH and wound infection or leakage. In 6 patients 30-day mortality could not be determined because of a shorter follow-up.* ^*d*^*Fisher-test*

## Discussion

In this study, the literature review showed that additional contralateral surgery after unilateral drainage in patients with a bilateral cSDH, was required in only 14% of cases. In our own, single center cohort study, bilateral surgery had a higher risk of surgical complications, but in cases with clinical equipoise for surgical approach, a unilateral or bilateral approach did not affect outcome.

The findings of this study indicate that additional contralateral treatment of a non-evacuated cSDH occurs only in a minority of patients. Naturally, the rate of additional contralateral treatment depends on the threshold for initial bilateral surgery of individual neurosurgeons. A recent study by Sundblom et al., in which the default surgical approach was unilateral and additional contralateral treatment was performed only if necessary, confirmed this, showing a contralateral treatment rate of 41% [23]. The fact that the contralateral treatment rate of Sundblom’s study is relatively high compared to the findings of our study, shows the added value of pre-operative selection of suitable candidates for surgical approach. Thus, given the significance of pre-operative selection and the relatively low incidence of additional contralateral surgery, it can be generally advised that a unilateral surgical approach mostly benefits patients in whom only one hematoma is large and symptomatic.

This study demonstrates that drainage of large ipsilateral hematomas is not associated with additional contralateral treatment, contrary to previous studies, which hypothesized that unilateral drainage in patients with a large ipsilateral hematomas would provoke contralateral hematoma growth as the result of decreased post-operative intracranial pressure and ipsilateral displacement of the brain [5, 6]. Scheichel et al. (2018) were the first to quantify the possible interaction between the two hematomas and the impact on outcome using the volume relation ratio. They suggest that bilateral evacuation is indicated when the ratio exceeds 0.40. This signifies that a larger contralateral hematoma volume and smaller ipsilateral hematoma volume would increase the ratio. This observation is in concordance with results of this study, indicating that a smaller surgical hematoma is a protective factor for additional contralateral treatment. A possible explanation could be that smaller ipsilateral hematomas, provide less counter pressure to its contralateral counterpart. Which, in turn, could lead to larger contralateral hematoma volumes and thus contralateral treatment. The fact that a larger contralateral hematoma volume is a risk factor for additional surgical treatment, is similar to what is known for patients treated with initial conservative therapy. For these patients, studies have shown that larger hematoma volume is a risk factor for eventual surgery [24, 25]. To understand the relation between unilateral drainage and intracranial cerebrospinal fluid dynamics more thoroughly, more research is warranted [26].

Our study showed a similar complication rate when there is clincial equipoise concerning surgical approach, but a significantly lower complication rate in all patients treated with unilateral surgery, compared to a bilateral approach. We included surgical complications only (specific to surgery side), since other, general complications (e.g. urinary tract infections, pneumonia, meningitis, delirium, thromboembolic events or electrolyte disturbances), are affected by other factors such as hospital admittance and patient frailty, rather than surgical approach [12, 27]. Several studies have investigated the effect of surgical approach on complication rate and found no difference [1, 3, 28]. However, none of these studies made a distinction between surgical and general complications, which precludes a fair comparison. Previous studies report inconsistent findings with regard to difference in reoperation rates between initial uni- or bilateral surgery. Some studies report no difference, with reoperation rates ranging from 14 to 22% for both surgical approaches [1, 3], whereas other studies report a significantly higher reoperation rate in patients treated with unilateral surgery [5, 6, 29]. Andersen-Ranberg et al. reported a reoperation rate of 28.7% for unilateral surgery and 14.1% for bilateral surgery, but the results of this study cannot be extrapolated to the current care as approximately 90% of all patients did not receive post-operative drainage [7]. In a study by Zolfaghari et al. the reoperation rate was also significantly higher in patients treated with unilateral surgery (18% vs. 32%), however, the majority (66.3%) of patients treated with unilateral surgery received a mini craniotomy, while almost all (90.2%) patients treated with bilateral surgery received burr hole craniostomy [29]. Comparing both groups is therefore questionable since standard surgical treatment is burr hole craniostomy [30–33].

Based on the findings in this cohort, it may be argued that a unilateral surgical approach is feasible for certain bilateral cSDH patients, although future prospective studies are necessary to define in which patients unilateral surgery may suffice. Patients receiving upfront bilateral surgery could be more severely affected, and their smallest hematomas might be larger than those of patients receiving initial unilateral surgery, which could lead to worse outcome. However, in the group with clinical equipoise, patients receiving unilateral surgery had a higher Markwalder Grading Scale (which indicates severity of symptoms) compared to patients receiving bilateral surgery. The same applied for amount and extent of midline shift, as patients who underwent unilateral surgery had both a higher incidence and greater extent of midline shift, than those treated with bilateral surgery. Volume of the smallest hematoma was larger for patients treated with unilateral surgery on the other hand. Due to the relatively small sample size, it was not possible to statistically adjust for these discrepancies. Despite this, the implementation of the neurosurgical panel to identify patients in whom treatment variation exists, significantly reduced our risk of bias, as it accurately reflects real-world clinical practice and accounts for the absence of definitive criteria for either unilateral or bilateral surgical drainage. These considerations support the notion that a less invasive, unilateral, approach could achieve similar outcomes. However, this is the first study to incorporate a subgroup with patients in whom there is clinical equipoise with regard to surgical approach, and the sample size is limited.

### Limitations and strengths

In the systematic review, not all studies could be integrated into the meta-analysis. Therefore, the sample sizes remained small, indicating that larger, additional studies, are required. Nevertheless, this is the first study to integrate all findings on previous research concerning surgical approach for bilateral cSDH, making it the most detailed analysis on this topic currently available. In our own cohort study, similar, sample size related limitations apply. For example, the sample sizes of the contralateral treatment group and the subgroups of the clinical equipoise group and the propensity score-matched cohort were relatively small. This limits statistical power and the generalizability of our results. Further limitations of the cohort study include its’ retrospective nature and performance at one single institution. However, all consecutive cSDH patients between 2010 and 2022 in the study institution were identified, so selection bias is not looming. Moreover, surgical approach and reoperation rates could have been affected by individual surgeon preference. Yet, the implementation the two subgroups and their similar results add to the robustness of our results, marking it a significant strength.

## Conclusion

In patients with bilateral cSDH, additional contralateral treatment following initial unilateral surgery is required in 14% of all patients. In our own cohort, initial bilateral surgery carried a greater risk of surgical complications. However, in patients for whom clinical equipoise is presumed with regard to surgical approach and after adjustment for baseline clinical and radiological differences, initial unilateral or bilateral surgery did not affect outcome. Therefore, a unilateral surgical approach may be considered for selected patients with a bilateral cSDH. Future prospective studies are required to define exactly in which patients unilateral surgery may suffice.

## Electronic supplementary material

Below is the link to the electronic supplementary material.

Supplementary Material 1

Supplementary Material 2

Supplementary Material 3

Supplementary Material 4

## Data Availability

No datasets were generated or analysed during the current study.

## References

[1] Zhang JJY et al (2020) Development of a prognostic scoring system to predict risk of reoperation for contralateral hematoma growth after unilateral evacuation of bilateral chronic subdural hematoma. J Clin Neurosci 78:79–85 32616352 10.1016/j.jocn.2020.06.009

[2] Lee J, Park JH (2014) Clinical characteristics of bilateral versus unilateral chronic subdural hematoma. Korean J Neurotrauma 10(2):49–54 27169033 10.13004/kjnt.2014.10.2.49 PMC4852599

[3] Motiei-Langroudi R et al (2019) Factors predicting the need for surgery of the opposite side after unilateral evacuation of bilateral chronic subdural hematomas. Neurosurgery 85(5):648–655 30265326 10.1093/neuros/nyy432

[4] Tsai TH et al (2010) A comparative study of the patients with bilateral or unilateral chronic subdural hematoma: precipitating factors and postoperative outcomes. J Trauma 68(3):571–575 20065879 10.1097/TA.0b013e3181a5f31c

[5] Scheichel F et al (2018) Contralateral progression after unilateral evacuation of bilateral chronic subdural hematomas: the volume relation ratio as prognostic factor? J Neurosurg 131(4):1227–1234 10.3171/2018.6.JNS18467 30497151

[6] Andersen-Ranberg NC et al (2017) Bilateral chronic subdural hematoma: unilateral or bilateral drainage? J Neurosurg 126(6):1905–1911 27392267 10.3171/2016.4.JNS152642

[7] Ronn Jensen TS et al (2018) [National guidelines for treatment of chronic subdural haematoma]. Ugeskr Laeger 180(42):V03180160 30327087

[8] Fujitani S et al (2017) Factors predicting contralateral hematoma growth after unilateral drainage of bilateral chronic subdural hematoma. J Neurosurg 126(3):755–759 27081904 10.3171/2016.1.JNS152655

[9] Sadrolhefazi A, Bloomfield SM (2000) Interhemispheric and bilateral chronic subdural hematoma. Neurosurg Clin N Am 11(3):455–463 10918015

[10] Chen FM et al (2020) Predictors of acute intracranial hemorrhage and recurrence of chronic subdural hematoma following burr hole drainage. BMC Neurol 20(1):92 32169039 10.1186/s12883-020-01669-5 PMC7069197

[11] Pang CH et al (2015) Acute intracranial bleeding and recurrence after Bur hole craniostomy for chronic subdural hematoma. J Neurosurg 123(1):65–74 25679282 10.3171/2014.12.JNS141189

[12] Rohde V, Graf G, Hassler W (2002) Complications of burr-hole craniostomy and closed-system drainage for chronic subdural hematomas: a retrospective analysis of 376 patients. Neurosurg Rev 25(1–2):89–94 11954771 10.1007/s101430100182

[13] Page MJ et al (2021) The PRISMA 2020 statement: an updated guideline for reporting systematic reviews. BMJ 372:n71 33782057 10.1136/bmj.n71 PMC8005924

[14] Wells GA, O’Connell BSD, Peterson J, Welch V, Losos M P Tugwell,. *The Newcastle-Ottawa Scale (NOS) for assessing the quality of nonrandomised studies in meta-analyses*. [cited 2022 December]; Available from: https://www.ohri.ca/programs/clinical_epidemiology/oxford.asp

[15] *Improving the Outcome of Chronic Subdural Hematoma by Embolization of Middle Meningeal Artery (ELIMINATE)*. (2023) [cited 2023 08-09-2023]; Available from: https://clinicaltrials.gov/study/NCT04511572?cond=chronic%20subdural%20hematoma&term=eliminate&rank=1

[16] Immenga S et al (2022) Tranexamic acid to prevent operation in chronic subdural haematoma (TORCH): study protocol for a randomised placebo-controlled clinical trial. Trials 23(1):56 35042560 10.1186/s13063-021-05907-0 PMC8767703

[17] https://www.castoredc.com/. (2024)

[18] Higgins JP et al (2003) Measuring inconsistency in meta-analyses. BMJ 327(7414):557–560 12958120 10.1136/bmj.327.7414.557 PMC192859

[19] Balduzzi S, Rücker G, Schwarzer G (2019) How to perform a meta-analysis with R: a practical tutorial. Evid Based Ment Health 22(4):153–160 31563865 10.1136/ebmental-2019-300117 PMC10231495

[20] Takahashi S et al (2018) Proposal of treatment strategies for bilateral chronic subdural hematoma based on laterality of treated hematoma. Asian J Neurosurg 13(4):1134–1139 30459882 10.4103/ajns.AJNS_124_18 PMC6208213

[21] Shen J et al (2019) Risk factors for contralateral hematoma progression after unilateral evacuation of bilateral chronic subdural hematomas. World Neurosurg 126:e773–e778 30853519 10.1016/j.wneu.2019.02.148

[22] Blaauw J et al (2020) Neurosurgical and perioperative management of chronic subdural hematoma. Front Neurol 11:550 32636797 10.3389/fneur.2020.00550 PMC7317017

[23] Sundblom J, Sandberg E, Ronne-Engstrom E (2022) Trauma mechanisms and surgical outcomes in the elderly patient with chronic subdural hematoma. Can Geriatr J 25(1):40–48 35310470 10.5770/cgj.25.519 PMC8887704

[24] Wang D et al (2022) Risk factor analysis of the Conservative treatment in chronic subdural hematomas: A substudy of the ATOCH trial. Adv Ther 39(4):1630–1641 35133631 10.1007/s12325-022-02057-w

[25] Zhang X et al (2023) Factors influencing wait-and-watch management in mild primary chronic subdural hematoma: a retrospective case-control study. Acta Neurol Belg 123(6):2277–2286 37269419 10.1007/s13760-023-02293-z

[26] Chen JW (2019) Commentary: factors predicting the need for surgery of the opposite side after unilateral evacuation of bilateral chronic subdural hematomas. Neurosurgery 85(5):E835–e836 30307513 10.1093/neuros/nyy443

[27] Mori K, Maeda M (2001) Surgical treatment of chronic subdural hematoma in 500 consecutive cases: clinical characteristics, surgical outcome, complications, and recurrence rate. Neurol Med Chir (Tokyo) 41(8):371–381 11561347 10.2176/nmc.41.371

[28] Kerttula S et al (2022) The effect of antithrombotic therapy on the recurrence and outcome of chronic subdural hematoma after burr-hole craniostomy in a population-based cohort. Acta Neurochir (Wien) 164(10):2699–2708 35972559 10.1007/s00701-022-05337-0 PMC9519695

[29] Zolfaghari S et al (2021) Risk factors for need of reoperation in bilateral chronic subdural haematomas. Acta Neurochir (Wien) 163(7):1849–1856 33796888 10.1007/s00701-021-04811-5 PMC8195919

[30] Henry J et al (2022) Management of chronic subdural hematoma: A systematic review and component network Meta-analysis of 455 studies with 103 645 cases. Neurosurgery 91(6):842–855 36170165 10.1227/neu.0000000000002144

[31] Liu W, Bakker NA, Groen RJ (2014) Chronic subdural hematoma: a systematic review and meta-analysis of surgical procedures. J Neurosurg 121(3):665–673 24995782 10.3171/2014.5.JNS132715

[32] Abecassis IJ, Kim LJ (2017) Craniotomy for treatment of chronic subdural hematoma. Neurosurg Clin N Am 28(2):229–237 28325457 10.1016/j.nec.2016.11.005

[33] Zolfaghari S et al (2021) Burr hole craniostomy versus minicraniotomy in chronic subdural hematoma: a comparative cohort study. Acta Neurochir (Wien) 163(11):3217–3223 34328561 10.1007/s00701-021-04902-3 PMC8520513

