# Supplementary material for: Unilateral or bilateral drainage for patients with bilateral chronic subdural hematoma: a systematic review and retrospective cohort study
Source: Neurosurg Rev. 2025 May 6;48(1):403. doi: 10.1007/s10143-025-03530-0 (PMC12053184; doi:10.1007/s10143-025-03530-0)
Supplement: Supplementary file 1 — Supplementary Material 1 [file 10143_2025_3530_MOESM1_ESM.docx]

# Supplement 1: Online search strategy

**Table 1**

| Database(s): Ovid MEDLINE(R) ALL 1946 to May 29, 2024 | |  |
| --- | --- | --- |
| Search Strategy: | |  |
| # | Searches | Results |
| 1 | ((bilateral or "Double side*" or contralateral) adj3 ((chronic or subacute) adj3 subdural adj3 (h?ematoma* or h?emorrhage* or bleed* or blood*))).ti,ab,kf. | 244 |
| 2 | ((bilateral or "double side*" or contralateral) adj3 (cSDH or SDH)).ti,ab,kf. | 145 |
| 3 | or/1-2 | 361 |
| 4 | exp General Surgery/ or exp Recurrence/ or exp Patient Readmission/ or exp Postoperative Complications/ | 868578 |
| 5 | (surger* or surgical* or repair* or intervention* or procedure* or operat* or burr hole or craniostom or twist drill or craniotomy or recurrence or rehospitali* or readmission or reoperat*).ti,ab,kf. | 5710557 |
| 6 | ((cranial or cranio or skull or unilateral or bilateral) adj3 (drain* or decompress*)).ti,ab,kf. | 1978 |
| 7 | (complication adj3 (postoperati* or postsurger* or postsurgical*)).ti,ab,kf. | 17323 |
| 8 | or/4-7 | 6065284 |
| 9 | 3 and 8 | 239 |

*MEDLINE search*

**Table 2**

| *Database(s): Embase Classic+Embase 1947 to 2024 May 29* | |  |
| --- | --- | --- |
| *Search Strategy:* | |  |
| *#* | *Searches* | *Results* |
| *1* | *((bilateral or "Double side*" or contralateral) adj3 ((chronic or subacute) adj3 subdural adj3 (h?ematoma* or h?emorrhage* or bleed* or blood*))).ti,ab,kf.* | *293* |
| *2* | *((bilateral or "double side*" or contralateral) adj3 (cSDH or SDH)).ti,ab,kf.* | *200* |
| *3* | *or/1-2* | *460* |
| *4* | *exp surgery/ or exp recurrent disease/ or exp hospital readmission/ or exp postoperative complication/* | *6843443* |
| *5* | *(surger* or surgical* or repair* or intervention* or procedure* or operat* or burr hole or craniostom or twist drill or craniotomy or recurrence or rehospitali* or readmission or reoperat*).ti,ab,kf.* | *7909032* |
| *6* | *((cranial or cranio or skull or unilateral or bilateral) adj3 (drain* or decompress*)).ti,ab,kf.* | *2850* |
| *7* | *(complication adj3 (postoperati* or postsurger* or postsurgical*)).ti,ab,kf.* | *26422* |
| *8* | *or/4-7* | *11075102* |
| *9* | *3 and 8* | *358* |

*EMBASE search*

**Table 3**

| *Date Run:* | *30-5-2024 12:51* |  |
| --- | --- | --- |
|  |  |  |
| *ID* | *Search* | *Hits* |
| *#1* | *((bilateral or (double near/2 side*) or contralateral) near/3 ((chronic or subacute) near/3 subdural near/3 (h?ematoma* or h?emorrhage* or bleed* or blood*)))* | *9* |
| *#2* | *((bilateral or (double near/2 side*) or contralateral) near/3 (cSDH or SDH))* | *12* |
| *#3* | *{or #1-#2}* | *20* |
| *#4* | *MeSH descriptor: [General Surgery] explode all trees* | *506* |
| *#5* | *MeSH descriptor: [Recurrence] explode all trees* | *16533* |
| *#6* | *MeSH descriptor: [Patient Readmission] explode all trees* | *1614* |
| *#7* | *MeSH descriptor: [Postoperative Complications] explode all trees* | *55136* |
| *#8* | *(surger* or surgical* or repair* or intervention* or procedure* or operat* or "burr hole" or craniostom or "twist drill" or craniotomy or recurrence or rehospitali* or readmission or reoperat*)* | *1088361* |
| *#9* | *((cranial or cranio or skull or unilateral or bilateral) near/3 (drain* or decompress*))* | *162* |
| *#10* | *(complication near/3 (postoperati* or postsurger* or postsurgical*))* | *20958* |
| *#11* | *{or #4-#10}* | *1093128* |
| *#12* | *#3 AND #11* | *20* |

*Cochrane Library search*

**Table 4**

| *TITLE-ABS-KEY-AUTH ( ( ( bilateral OR "double side*" OR contralateral ) W/3 ( ( chronic OR subacute ) W/3 subdural W/3 ( h?ematoma* OR h?emorrhage* OR bleed* OR blood* ) ) ) OR ( ( bilateral OR "double side*" OR contralateral ) W/3 ( csdh OR sdh ) ) ) AND TITLE-ABS-KEY-AUTH ( ( surger* OR surgical* OR repair* OR intervention* OR procedure* OR operat* OR "burr hole" OR craniostom OR "twist drill" OR craniotomy OR recurrence OR rehospitali* OR readmission OR reoperat* ) OR ( ( cranial OR cranio OR skull OR unilateral OR bilateral ) W/3 ( drain* OR decompress* ) ) OR ( complication W/3 ( postoperati* OR postsurger* OR postsurgical* ) ) )* | *156* |
| --- | --- |

*SCOPUS search*

**Table 5**

| *Thu, May 30, 2024 00:26:31 p.m, Database - CINAHL Plus with Full Tekst* | |  |
| --- | --- | --- |
| *#* | *Query* | *Results* |
| *S12* | *S3 AND S11* | *51* |
| *S11* | *S4 OR S5 or S6 or S7 or S8 or S9 or S10* | *2,677,719* |
| *S10* | *TX (complication N3 (postoperati* or postsurger* or postsurgical*))* | *102,443* |
| *S9* | *TX ((cranial or cranio or skull or unilateral or bilateral) N3 (drain* or decompress*))* | *713* |
| *S8* | *TX (surger* or surgical* or repair* or intervention* or procedure* or operat* or "burr hole" or craniostom or "twist drill" or craniotomy or recurrence or rehospitali* or readmission or reoperat*)* | *2,482,325* |
| *S7* | *(MH "Postoperative Complications+")* | *140,081* |
| *S6* | *(MM "Recurrence")* | *12,708* |
| *S5* | *(MM "Readmission")* | *8,268* |
| *S4* | *(MH "Surgery, Operative+")* | *771,611* |
| *S3* | *S1 or S2* | *60* |
| *S2* | *TX ((bilateral or "double side*" or contralateral) N3 (cSDH or SDH))* | *48* |
| *S1* | *TX ((bilateral or "Double side*" or contralateral) N3 ((chronic or subacute) N3 subdural N3 (h?ematoma* or h?emorrhage* or bleed* or blood*)))* | *14* |

*CINAHL search*
