# Supplementary material for: Unilateral or bilateral drainage for patients with bilateral chronic subdural hematoma: a systematic review and retrospective cohort study
Source: Neurosurg Rev. 2025 May 6;48(1):403. doi: 10.1007/s10143-025-03530-0 (PMC12053184; doi:10.1007/s10143-025-03530-0)
Supplement: Supplementary file 2 — Supplementary Material 2 [file 10143_2025_3530_MOESM2_ESM.docx]

**Figure 1**
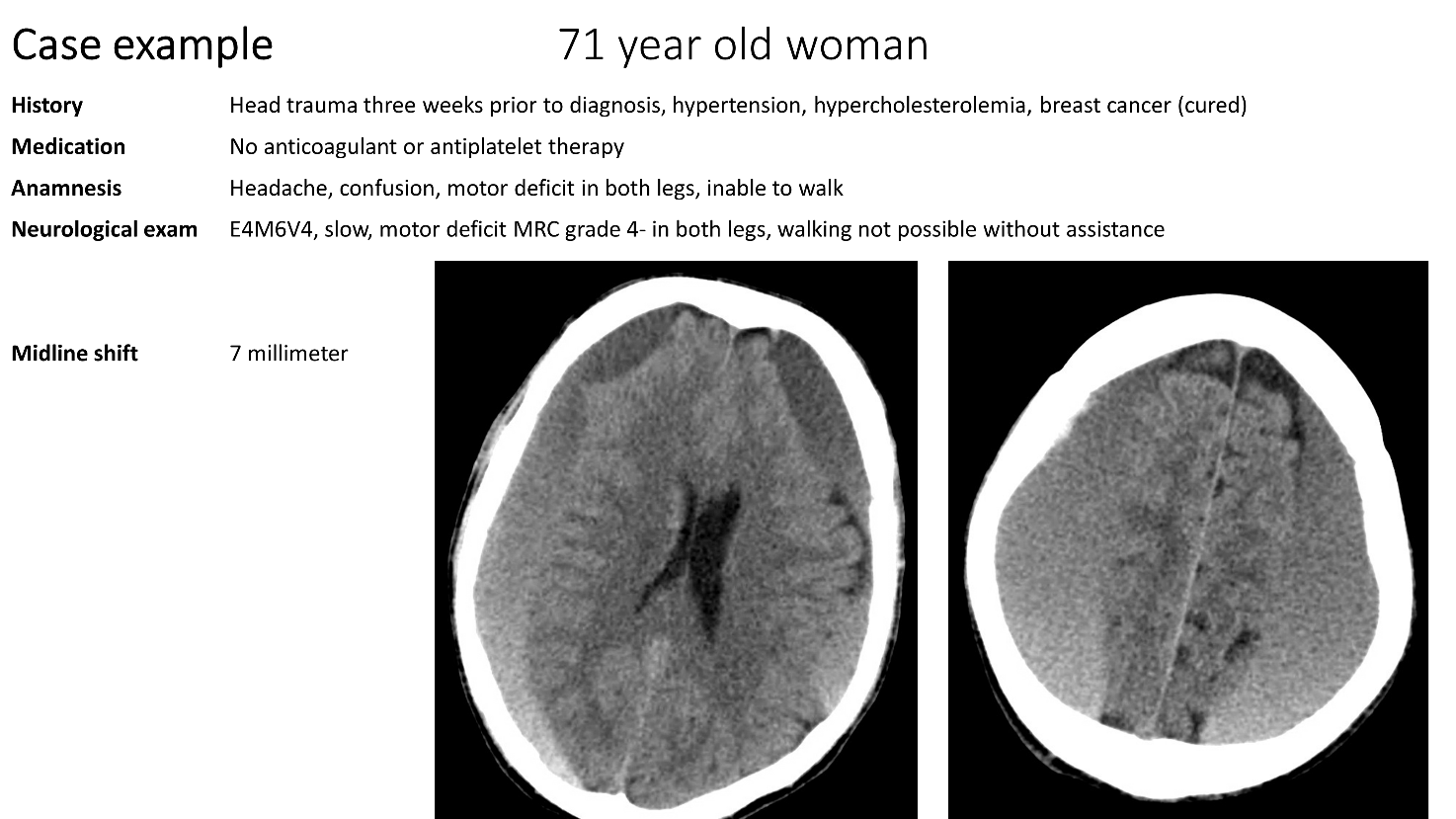


*Case presentation example (fictive case).*

| **Table 1. Per case surgical approach decision** | | | | |
| --- | --- | --- | --- | --- |
| **Case** | **Adjudicator 1** | **Adjudicator 2** | **Adjudicator 3** | **Equipoise** |
| 1 | Bilateral surgery | Bilateral surgery | Bilateral surgery | No |
| 2 | Unilateral surgery | Unilateral surgery | Unilateral surgery | No |
| 3 | Bilateral surgery | Unilateral surgery | Bilateral surgery | Yes |
| 4 | Bilateral surgery | Bilateral surgery | Bilateral surgery | No |
| 5 | Bilateral surgery | Unilateral surgery | Bilateral surgery | Yes |
| 6 | Bilateral surgery | Bilateral surgery | Bilateral surgery | No |
| 7 | Bilateral surgery | Unilateral surgery | Uni- or bilateral surgery | Yes |
| 8 | Bilateral surgery | Uni- or bilateral surgery | Bilateral surgery | Yes |
| 9 | Unilateral surgery | Unilateral surgery | Unilateral surgery | No |
| 10 | Unilateral surgery | Unilateral surgery | Unilateral surgery | No |
| 11 | Bilateral surgery | Bilateral surgery | Bilateral surgery | No |
| 12 | Bilateral surgery | Uni- or bilateral surgery | Uni- or bilateral surgery | Yes |
| 13 | Uni- or bilateral surgery | Unilateral surgery | Unilateral surgery | Yes |
| 14 | Uni- or bilateral surgery | Unilateral surgery | Unilateral surgery | Yes |
| 15 | Unilateral surgery | Unilateral surgery | Unilateral surgery | No |
| 16 | Unilateral surgery | Unilateral surgery | Unilateral surgery | No |
| 17 | CT not available | CT not available | CT not available | N.A. |
| 18 | Bilateral surgery | Bilateral surgery | Bilateral surgery | No |
| 19 | Unilateral surgery | Unilateral surgery | Uni- or bilateral surgery | Yes |
| 20 | Bilateral surgery | Uni- or bilateral surgery | Bilateral surgery | Yes |
| 21 | Uni- or bilateral surgery | Unilateral surgery | Bilateral surgery | Yes |
| 22 | Unilateral surgery | Unilateral surgery | Unilateral surgery | No |
| 23 | Uni- or bilateral surgery | Unilateral surgery | Uni- or bilateral surgery | Yes |
| 24 | Bilateral surgery | Unilateral surgery | Bilateral surgery | Yes |
| 25 | Unilateral surgery | Unilateral surgery | Unilateral surgery | No |
| 26 | Unilateral surgery | Unilateral surgery | Unilateral surgery | No |
| 27 | Bilateral surgery | Uni- or bilateral surgery | Bilateral surgery | Yes |
| 28 | Uni- or bilateral surgery | Unilateral surgery | Unilateral surgery | Yes |
| 29 | Unilateral surgery | Unilateral surgery | Unilateral surgery | No |
| 30 | Uni- or bilateral surgery | Unilateral surgery | Unilateral surgery | Yes |
| 31 | Unilateral surgery | Unilateral surgery | Unilateral surgery | No |
| 32 | Unilateral surgery | Unilateral surgery | Unilateral surgery | No |
| 33 | Uni- or bilateral surgery | Bilateral surgery | Bilateral surgery | Yes |
| 34 | Uni- or bilateral surgery | Bilateral surgery | Bilateral surgery | Yes |
| 35 | Unilateral surgery | Unilateral surgery | Bilateral surgery | Yes |
| 36 | Uni- or bilateral surgery | Bilateral surgery | Bilateral surgery | Yes |
| 37 | Unilateral surgery | Unilateral surgery | Unilateral surgery | No |
| 38 | Unilateral surgery | Unilateral surgery | Uni- or bilateral surgery | Yes |
| 39 | Uni- or bilateral surgery | Unilateral surgery | Bilateral surgery | Yes |
| 40 | Uni- or bilateral surgery | Unilateral surgery | Uni- or bilateral surgery | Yes |
| 41 | Unilateral surgery | Uni- or bilateral surgery | Uni- or bilateral surgery | Yes |
| 42 | Unilateral surgery | Unilateral surgery | Unilateral surgery | No |
| 43 | Unilateral surgery | Unilateral surgery | Unilateral surgery | No |
| 44 | Uni- or bilateral surgery | Unilateral surgery | Bilateral surgery | Yes |
| 45 | Unilateral surgery | Unilateral surgery | Unilateral surgery | No |
| 46 | Unilateral surgery | Unilateral surgery | Unilateral surgery | No |
| 47 | Unilateral surgery | Unilateral surgery | Unilateral surgery | No |
| 48 | Bilateral surgery | Unilateral surgery | Uni- or bilateral surgery | Yes |
| 49 | Unilateral surgery | Unilateral surgery | Unilateral surgery | No |
| 50 | Uni- or bilateral surgery | Unilateral surgery | Bilateral surgery | Yes |
| 51 | Unilateral surgery | Unilateral surgery | Unilateral surgery | No |
| 52 | Unilateral surgery | Unilateral surgery | Unilateral surgery | No |
| 53 | Unilateral surgery | Unilateral surgery | Uni- or bilateral surgery | Yes |
| 54 | Bilateral surgery | Bilateral surgery | Bilateral surgery | No |
| 55 | Unilateral surgery | Unilateral surgery | Unilateral surgery | No |
| 56 | Unilateral surgery | Unilateral surgery | Unilateral surgery | No |
| 57 | Uni- or bilateral surgery | Bilateral surgery | Uni- or bilateral surgery | Yes |
| 58 | Uni- or bilateral surgery | Unilateral surgery | Unilateral surgery | Yes |
| 59 | Uni- or bilateral surgery | Unilateral surgery | Bilateral surgery | Yes |
| 60 | Bilateral surgery | Unilateral surgery | Bilateral surgery | Yes |
| 61 | Unilateral surgery | Unilateral surgery | Unilateral surgery | No |
| 62 | Unilateral surgery | Unilateral surgery | Unilateral surgery | No |
| 63 | Unilateral surgery | Unilateral surgery | Unilateral surgery | No |
| 64 | Bilateral surgery | Unilateral surgery | Uni- or bilateral surgery | Yes |
| 65 | Unilateral surgery | Unilateral surgery | Unilateral surgery | No |
| 66 | Uni- or bilateral surgery | Unilateral surgery | Bilateral surgery | Yes |
| 67 | Bilateral surgery | Unilateral surgery | Bilateral surgery | Yes |
| 68 | Unilateral surgery | Unilateral surgery | Uni- or bilateral surgery | Yes |
| 69 | Uni- or bilateral surgery | Unilateral surgery | Bilateral surgery | Yes |
| 70 | Uni- or bilateral surgery | Unilateral surgery | Unilateral surgery | Yes |
| 71 | Unilateral surgery | Uni- or bilateral surgery | Bilateral surgery | Yes |
| 72 | Uni- or bilateral surgery | Bilateral surgery | Bilateral surgery | Yes |
| 73 | Uni- or bilateral surgery | Unilateral surgery | Bilateral surgery | Yes |
| 74 | Bilateral surgery | Bilateral surgery | Bilateral surgery | No |
| 75 | Bilateral surgery | Bilateral surgery | Bilateral surgery | No |
| 76 | Unilateral surgery | Unilateral surgery | Uni- or bilateral surgery | Yes |
| 77 | Bilateral surgery | Unilateral surgery | Bilateral surgery | Yes |
| 78 | Uni- or bilateral surgery | Unilateral surgery | Bilateral surgery | Yes |
| 79 | Unilateral surgery | Unilateral surgery | Unilateral surgery | No |
| 80 | Unilateral surgery | Unilateral surgery | Unilateral surgery | No |
| 81 | Bilateral surgery | Bilateral surgery | Bilateral surgery | No |
| 82 | Uni- or bilateral surgery | Uni- or bilateral surgery | Bilateral surgery | Yes |
| 83 | Bilateral surgery | Unilateral surgery | Uni- or bilateral surgery | Yes |
| 84 | Unilateral surgery |  | Unilateral surgery | Yes |
| 85 | Unilateral surgery | Unilateral surgery | Uni- or bilateral surgery | Yes |
| 86 | Unilateral surgery | Bilateral surgery | Unilateral surgery | Yes |
| 87 | Uni- or bilateral surgery | Unilateral surgery | Uni- or bilateral surgery | Yes |
| 88 | Uni- or bilateral surgery | Unilateral surgery | Uni- or bilateral surgery | Yes |
| 89 | Uni- or bilateral surgery | Unilateral surgery | Bilateral surgery | Yes |
| 90 | Unilateral surgery | Unilateral surgery | Bilateral surgery | Yes |
| 91 | Unilateral surgery | Unilateral surgery | Unilateral surgery | No |
| 92 | Unilateral surgery | Unilateral surgery | Uni- or bilateral surgery | Yes |
| 93 | Unilateral surgery | Unilateral surgery | Unilateral surgery | No |
| 94 | Uni- or bilateral surgery | Unilateral surgery | Uni- or bilateral surgery | Yes |
| 95 | Uni- or bilateral surgery | Unilateral surgery | Bilateral surgery | Yes |
| 96 | Unilateral surgery | Unilateral surgery | Unilateral surgery | No |
| 97 | Unilateral surgery | Unilateral surgery | Uni- or bilateral surgery | Yes |
| 98 | Bilateral surgery | Bilateral surgery | Bilateral surgery | No |
| 99 | Uni- or bilateral surgery | Unilateral surgery | Bilateral surgery | Yes |
| 100 | Bilateral surgery | Uni- or bilateral surgery | Bilateral surgery | Yes |
| 101 | Unilateral surgery | Unilateral surgery | Uni- or bilateral surgery | Yes |
| 102 | Bilateral surgery | Unilateral surgery | Bilateral surgery | Yes |
| 103 | Unilateral surgery | Unilateral surgery | Unilateral surgery | No |
| 104 | Uni- or bilateral surgery | Bilateral surgery | Bilateral surgery | Yes |
| 105 | Unilateral surgery | Unilateral surgery | Unilateral surgery | No |
| 106 | Uni- or bilateral surgery | Unilateral surgery | Uni- or bilateral surgery | Yes |
| 107 | Unilateral surgery | Unilateral surgery | Unilateral surgery | No |
| 108 | Uni- or bilateral surgery | Unilateral surgery | Bilateral surgery | Yes |
| 109 | Bilateral surgery | Bilateral surgery | Bilateral surgery | No |
| 110 | Unilateral surgery | Unilateral surgery | Uni- or bilateral surgery | Yes |
| 111 | Unilateral surgery | Unilateral surgery | Unilateral surgery | No |
| 112 | Bilateral surgery | Bilateral surgery | Bilateral surgery | No |
| 113 | Uni- or bilateral surgery | Unilateral surgery | Uni- or bilateral surgery | Yes |
| 114 | Unilateral surgery | Unilateral surgery | Unilateral surgery | No |
| 115 | Bilateral surgery | Unilateral surgery | Bilateral surgery | Yes |
| 116 | Uni- or bilateral surgery | Unilateral surgery | Uni- or bilateral surgery | Yes |
| 117 | Uni- or bilateral surgery | Unilateral surgery | Bilateral surgery | Yes |
| 118 | Uni- or bilateral surgery | Unilateral surgery | Bilateral surgery | Yes |
| 119 | Uni- or bilateral surgery | Bilateral surgery | Bilateral surgery | Yes |
| 120 | Unilateral surgery | Unilateral surgery | Unilateral surgery | No |
| 121 | Unilateral surgery | Unilateral surgery | Unilateral surgery | No |
| 122 | Unilateral surgery | Unilateral surgery | Bilateral surgery | Yes |
| 123 | Unilateral surgery | Unilateral surgery | Unilateral surgery | No |
| 124 | Unilateral surgery | Unilateral surgery | Uni- or bilateral surgery | Yes |
| 125 | Uni- or bilateral surgery | Unilateral surgery | Bilateral surgery | Yes |
| 126 | Unilateral surgery | Unilateral surgery | Unilateral surgery | No |
| 127 | Unilateral surgery | Unilateral surgery | Unilateral surgery | No |
| 128 | Unilateral surgery | Unilateral surgery | Unilateral surgery | No |
| 129 | Unilateral surgery | Unilateral surgery | Unilateral surgery | No |
| 130 | Unilateral surgery | Unilateral surgery | Unilateral surgery | No |
| 131 | Bilateral surgery | Unilateral surgery | Bilateral surgery | Yes |
| 132 | Uni- or bilateral surgery | Unilateral surgery | Uni- or bilateral surgery | Yes |
| 133 | Unilateral surgery | Unilateral surgery | Unilateral surgery | No |
| 134 | Uni- or bilateral surgery | Unilateral surgery | Bilateral surgery | Yes |
| 135 | Unilateral surgery | Unilateral surgery | Unilateral surgery | No |
| 136 | Unilateral surgery | Unilateral surgery | Unilateral surgery | No |
| 137 | Bilateral surgery | Uni- or bilateral surgery | Bilateral surgery | Yes |
| 138 | Bilateral surgery | Bilateral surgery | Bilateral surgery | No |
| 139 | Uni- or bilateral surgery | Unilateral surgery | Bilateral surgery | Yes |
| 140 | Unilateral surgery | Unilateral surgery | Unilateral surgery | No |
| 141 | Uni- or bilateral surgery | Unilateral surgery | Uni- or bilateral surgery | Yes |
| 142 | Bilateral surgery | Unilateral surgery | Bilateral surgery | Yes |
| 143 | Unilateral surgery | Unilateral surgery | Unilateral surgery | No |
| 144 | Uni- or bilateral surgery | Unilateral surgery | Uni- or bilateral surgery | Yes |
| 145 | Bilateral surgery | Unilateral surgery | Bilateral surgery | Yes |
| 146 | Unilateral surgery | Unilateral surgery | Bilateral surgery | Yes |
| 147 | Unilateral surgery | Uni- or bilateral surgery | Uni- or bilateral surgery | Yes |
| 148 | Uni- or bilateral surgery | Unilateral surgery | Uni- or bilateral surgery | Yes |
| 149 | Unilateral surgery | Unilateral surgery | Unilateral surgery | No |
| 150 | Unilateral surgery | Unilateral surgery | Unilateral surgery | No |
| 151 | Unilateral surgery | Unilateral surgery | Unilateral surgery | No |
| 152 | Bilateral surgery | Uni- or bilateral surgery | Bilateral surgery | Yes |
| 153 | Unilateral surgery | Unilateral surgery | Unilateral surgery | No |
| 154 | Bilateral surgery | Unilateral surgery | Uni- or bilateral surgery | Yes |
| 155 | Unilateral surgery | Unilateral surgery | Unilateral surgery | No |
| 156 | Bilateral surgery | Bilateral surgery | Bilateral surgery | No |
| 157 | Bilateral surgery | Unilateral surgery | Unilateral surgery | Yes |
| 158 | Bilateral surgery | Bilateral surgery | Bilateral surgery | No |
| 159 | Bilateral surgery | Unilateral surgery | Bilateral surgery | Yes |
| 160 | Unilateral surgery | Unilateral surgery | Unilateral surgery | No |
| 161 | Unilateral surgery | Unilateral surgery | Unilateral surgery | No |
| 162 | Unilateral surgery | Unilateral surgery | Unilateral surgery | No |
| 163 | Bilateral surgery | Bilateral surgery | Bilateral surgery | No |
| 164 | Unilateral surgery | Unilateral surgery | Unilateral surgery | No |
| 165 | Unilateral surgery | Unilateral surgery | Unilateral surgery | No |
| 166 | Unilateral surgery | Unilateral surgery | Unilateral surgery | No |
| 167 | Unilateral surgery | Unilateral surgery | Unilateral surgery | No |
| 168 | Uni- or bilateral surgery | Bilateral surgery | Bilateral surgery | Yes |
| 169 | Uni- or bilateral surgery | Unilateral surgery | Uni- or bilateral surgery | Yes |
| 170 | Unilateral surgery | Unilateral surgery | Unilateral surgery | No |
| 171 | Bilateral surgery | Bilateral surgery | Bilateral surgery | No |
| 172 | Unilateral surgery | Unilateral surgery | Unilateral surgery | No |
| 173 | Unilateral surgery | Unilateral surgery | Unilateral surgery | No |
| 174 | Bilateral surgery | Bilateral surgery | Bilateral surgery | No |
| 175 | Bilateral surgery | Bilateral surgery | Bilateral surgery | No |
| 176 | Unilateral surgery | Unilateral surgery | Unilateral surgery | No |
| 177 | Uni- or bilateral surgery | Bilateral surgery | Bilateral surgery | Yes |
| 178 | Bilateral surgery | Bilateral surgery | Bilateral surgery | No |
| **For case 17 imaging was not available. Therefore, clinical equipoise could not be assessed and this patient was excluded from the clinical equipoise group.CT: computed tomography, N.A.: not applicable.* | | | | |

| **Table 2. Baseline characteristics of 178 patients treated with initial bilateral or unilateral surgery** | | | |  | | |  |
| --- | --- | --- | --- | --- | --- | --- | --- |
| *Variable* | *Total (n=178)* | *Bilateral surgery (n=111)* | | | *Unilateral surgery (n=67)* | | *p-value* |
| Age (SD) | 73.1 (10.9) | 73.7 (10.4)  84 (75.7) | | | 72.1 (11.8)  49 (73.1) | | 0.366*^a^* |
| Male (%) | 133 (74.7) |  |  |  |  |  | 0.705*^b^* |
| *History* |  |  |  | |  |  |  |
| Arrhythmia (%) | 43 (24.2) | 23 (20.7)  21 (18.9)  23 (20.7)  3 (2.7)  12 (10.8)  24 (21.6)  45 (40.5)  27 (24.3)  10 (9.0)  56 (50.5)  0 (0)  38 (34.2)  64 (57.6)  3 (2.7) | | | 20 (29.9)  12 (17.9)  9 (13.4)  6 (9.0)  5 (7.5)  18 (26.9)  28 (41.8)  12 (17.9)  6 (9.0)  36 (53.7)  5 (7.6)  19 (28.8)  39 (59.1)  3 (4.5) | | 0.168^b^ |
| CVA (%) | 33 (18.5) |  |  |  |  |  | 0.867^b^ |
| Ischemic heart disease (%) | 32 (18.0) |  |  |  |  |  | 0.220*^b^* |
| VTE/PE (%) | 9 (5.1) |  |  |  |  |  | 0.083*^d^* |
| COPD (%) | 17 (9.4) |  |  |  |  |  | 0.462*^b^* |
| Diabetes mellitus (%) | 42 (23.6) |  |  |  |  |  | 0.425*^b^* |
| Hypertension (%) | 73 (41.0) |  |  |  |  |  | 0.869*^b^* |
| Malignancy (%) | 39 (21.9) |  |  |  |  |  | 0.316*^b^* |
| Alcoholism in history (%) | 16 (9.0) |  |  |  |  |  | 0.990*^b^* |
| *Medication* |  |  |  |  |  |  |  |
| Anticoagulant or antiplatelet therapy (%) | 92 (51.7) |  |  |  |  |  | 0.671^b^ |
| *Clinical features at diagnosis* |  |  |  |  |  |  |  |
| MGS (%)^171^ |  |  |  |  |  |  | 0.108^d^ |
| 0 | 5 (2.9) |  |  |  |  |  |  |
| 1 | 57 (33.0) |  |  |  |  |  |  |
| 2 | 103 (60.2) |  |  |  |  |  |  |
| 3 | 6 (3.5) |  |  |  |  |  |  |
| *Pre-operative radiological features* |  | 60 (54.1)  4.4 (2.8)  18.6 (6.6)  14.3 (5.5)  118.8 (44.2)  80.8 (35.8)  0.69 (0.20) | | | 61 (91.0)  8.6 (4.4)  20.1 (7.4)  9.8 (4.7)  127.9 (58.9)  37.4 (23.1)  0.30 (0.23) | |  |
| Midline shift (%)^177^ | 121 (68.4) |  |  |  |  |  | <0.001*^b^* |
| Midline shift in mm (SD)*^121^* | 6.8 (4.1) |  |  |  |  |  | <0.001*^a^* |
| Hematoma diameter largest hematoma in mm (SD)*^180^* | 19.1 (6.9) |  |  |  |  |  | 0.171*^a^* |
| Hematoma diameter smallest hematoma in mm (SD)*^180^* | 12.6 (5.7) |  |  |  |  |  | <0.001*^a^* |
| Hematoma volume largest hematoma in ml (SD)*^156^* | 122.1 (50.1) |  |  |  |  |  | 0.277*^a^* |
| Hematoma volume smallest hematoma in ml (SD)*^156^* | 65.0 (38.0) |  |  |  |  |  | <0.001*^a^* |
| Volume relation ratio (SD)*^156^* | 0.57 (0.27) |  |  |  |  |  | <0.001*^a^* |
| *With superscript in the column, ‘variable’ is indicated for how many patients data was available.CVA, cerebrovascular accident; VTE, ventous thrombolic embolism; PE, pulmonary embolism; COPD, chronic obstructive pulmonary disease; MGS, Markwalder Grading Scale. ^a^ Unpaired T-test, ^b^Chi-squared test, ^d^ Fisher’s exact test.* | | | | | | | |

| **Table 3. Characteristics of patients that received initial unilateral surgery** | | | |
| --- | --- | --- | --- |
| *Variable* | *Contralateral treatment (n=6)* | *No contralateral treatment (n=61)* | *p-value* |
| Age (SD) | 79.3 (6.1) | 71.4 (12.0) | 0.117*^a^* |
| Male (%) | 4 (66.7) | 45 (73.8) | 0.708*^d^* |
| *History* |  |  |  |
| Arrhythmia (%) | 4 (66.7) | 16 (26.2) | 0.039*^d^* |
| CVA (%) | 3 (50.0) | 9 (14.8) | 0.032*^d^* |
| Ischemic heart disease (%) | 1 (16.7) | 8 (13.1) | 0.808*^d^* |
| VTE (%) | 1 (16.7) | 5 (8.2) | 0.488*^d^* |
| COPD (%) | 0 (0) | 5 (8.2) | 0.466*^d^* |
| Diabetes mellitus (%) | 0 (0) | 18 (29.5) | 0.120*^d^* |
| Hypertension (%) | 2 (33.3) | 26 (42.6) | 0.660*^d^* |
| Malignancy (%) | 2 (33.3) | 10 (16.4) | 0.302*^d^* |
| Alcoholism in history (%) | 0 (0) | 6 (9.8) | 0.402*^d^* |
| *Medication* |  |  |  |
| Anticoagulant or antiplatelet therapy (%) | 5 (83.3) | 31 (50.8) | 0.127*^d^* |
| *Clinical features at diagnosis* |  |  |  |
| MGS (%)^66^ |  |  | 0.386*^d^* |
| 0 | 0 (0) | 5 (8.3) |  |
| 1 | 1 (16.7) | 18 (30.0) |  |
| 2 | 4 (66.7) | 35 (58.3) |  |
| 3 | 1 (16.7) | 2 (3.3) |  |
| *Pre-operative radiological features* |  |  |  |
| Midline shift (%) | 6 (100.0) | 55 (90.2) | 1.000*^d^* |
| Midline shift in mm (SD)*^61^* | 9.8 (8.5) | 8.5 (4.4) | 0.486*^a^* |
| Contralateral hematoma diameter in mm (IQR)^66^ | 9.0 (7.0-12.0) | 9.5 (6.0-11.3) | 0.642*^c^* |
| Ipsilateral hematoma diameter in mm (SD)^66^ | 16.2 (6.7) | 20.1 (7.4) | 0.227*^a^* |
| Contralateral hematoma volume in ml (SD)*^56^* | 61.4 (22.8) | 37.4 (23.1) | 0.014*^a^* |
| Ipsilateral hematoma volume in ml (SD)*^56^* | 140.1 (50.3) | 127.9 (58.9) | 0.633*^a^* |
| Volume relation ratio (SD)*^56^* | 0.48 (0.18) | 0.23 (0.23) | 0.172*^a^* |
| *Additional contralateral surgery was required in six patients (second column) and no further treatment was necessary in 61 patients (third column).With superscript in the column, ‘variable’ is indicated for how many patients data was available. ^a^ Unpaired T-test, ^b^Chi-squared test, ^c^ Mann Whitney U test, ^d^ Fisher’s exact test.* | | | |

| **Table 4. Outcomes in patient groups according to surgical approach** | | |  |
| --- | --- | --- | --- |
| *Outcome* | *Bilateral surgery (n=111)* | *Unilateral surgery (n=67)* | *p-value* |
| Reoperation (%) | 17 (15.3) | 13 (19.4) | 0.480*^a^* |
| Complications (%) | 11 (9.9) | 1 (1.5) | **0.032*^b^*** |
| Post-operative aSDH (%) | 4 (3.6) | 1 (1.5) | 0.651*^b^* |
| Post-operative wound (%) | 7 (6.3) | 0 (0) | **0.046*^b^*** |
| infection or leakage (%) |  |  |  |
| 30-day mortality^12^ (%) | 4 (3.9) | 2 (3.0) | 1.000*^b^* |
| *Complications are a composite of post-operative aSDH and wound infection or leakage. In 12 patients 30-day mortality could not be determined because of a shorter follow-up.* | | | |

**Description of the six cases requiring additional contralateral surgery**

**Case 1** – The patient presented with bilateral chronic subdural hematomas (cSDH), with a large isodense collection along the left convexity, causing significant mass effect and midline shift. Additionally, the patient exhibited paresis of the right leg. Consequently, unilateral surgery was performed on the left side. Postoperatively, the patient’s functional status remained unchanged compared to preoperative levels. Approximately one month later, the patient developed mental deterioration, increased somnolence, and gait disturbances. Subsequent imaging revealed a cSDH with mass effect and midline shift along the right convexity. This necessitated additional contralateral surgery.

**Case 2** – The patient presented with headache and mental deterioration. Imaging revealed bilateral cSDH, with a larger hematoma along the right convexity. Initial management consisted of unilateral surgery on the right side. However, four days later, the patient experienced neurological deterioration including indolence and right sided pronation of the arm. Imaging demonstrated an increase in the subdural collection along the left convexity, including acute components, as well as midline shift. Consequently, additional contralateral surgery was performed.

**Case 3** – The patient presented with progressive headache and somnolence over the past six weeks. Upon hospitalization, the patient experienced a decline in consciousness. Imaging revealed bilateral cSDH, with a larger hematoma along the left convexity, accompanied by midline shift. Consequently, unilateral surgery was performed on the left side. Two weeks after discharge, the patient developed progressive headaches and mental deterioration, including a decreased level of consciousness and bradyphrenia. Follow-up imaging demonstrated a cSDH along the right convexity with midline shift, necessitating additional contralateral surgery three weeks after the initial surgery.

**Case 4** – The patient was hospitalized with a urinary tract infection, headaches, nausea, and vomiting. One day later, the consciousness decreased. Imaging revealed bilateral cSDH. with a larger collection along the left convexity compared to the right, accompanied with midline shift. Unilateral surgery was performed on the left side. Two weeks later, the patient developed drowsiness, progressive apathy, and bradyphrenia. Imaging demonstrated bilateral cSDH with a small midline shift. Consequently, additional bilateral surgery was performed.

**Case 5** – The patient presented with progressive left-sided hemiparesis, headaches, and poverty of speech. Imaging revealed bilateral cSDH, with a larger collection along the right convexity, accompanied by midline shift. Additionally, a small hypodense subdural hematoma was observed on the left side. Unilateral surgery was performed for the hematoma on the right side. Postoperatively, there was significant improvement in left-sided motor function. However, approximately seven weeks later, the patient developed right-sided hemiparesis, gait disturbances, and progressive mental deterioration, including neurological confusion. Imaging demonstrated a significant increase in the cSDH along the left convexity, accompanied by a midline shift. Consequently, additional surgery was performed.

**Case 6** – The patient presented with headaches and memory complaints for the last few weeks. On the day of hospitalization, the patient suffered a head trauma, whereafter he experienced gait disturbance. There was a bilateral cSDH with a large collection along the right convexity causing significant midline shift. A smaller subdural collection was noted along the left convexity. Initial management involved unilateral surgery on the right side. Approximately two months later, the patient experienced mental deterioration including cognitive decline, and gait disturbances. On imaging there was an increase in the cSDH along the left convexity, accompanied by midline shift. This progression necessitated additional contralateral surgery.
