# Supplementary material for: Unilateral or bilateral drainage for patients with bilateral chronic subdural hematoma: a systematic review and retrospective cohort study
Source: Neurosurg Rev. 2025 May 6;48(1):403. doi: 10.1007/s10143-025-03530-0 (PMC12053184; doi:10.1007/s10143-025-03530-0)

**Supplement C Quality assessment**

| Table 1 Risk of bias and quality assessment for observational studies with NOS-Scale | | | | |
| --- | --- | --- | --- | --- |
| Study | **Selection** | **Comparability** | **Exposure** | **Quality** |
| Andersen-Ranberg et al., 2017 | ★★ |  | ★★★ | Poor |
| Fujitani et al., 2017 | ★★★★ | ★ | ★★★ | Good |
| Langroudi et al., 2018 | ★★★ | ★ | ★ | Poor |
| Scheichel et al., 2018 | ★★★ | ★ | ★★ | Good |
| Takahashi et al., 2018 | ★★★ | ★ | ★★ | Good |
| Shen et al., 2019* | ★★★ | ★ | ★★ | Good |
| Blaauw et al., 2020 | ★★★★ |  | ★ | Poor |
| Zhang et al., 2020 | ★★★★ | ★ | ★★ | Good |
| *The last column indicates AHRQ quality standards, * Maximum star allocation 8.* | | | | |

**Figure 1**


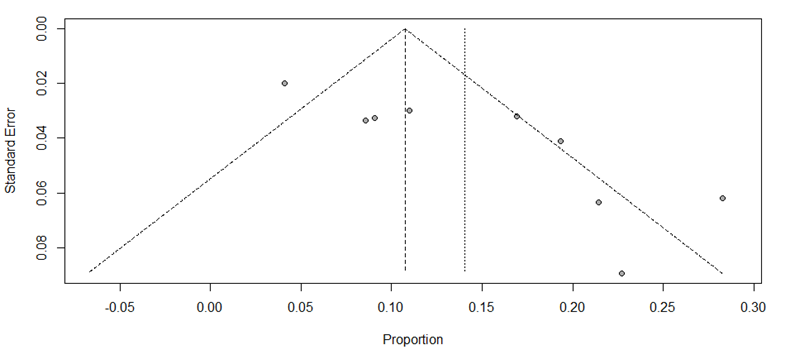

Supplement: Supplementary file 3 — Supplementary Material 3 [file 10143_2025_3530_MOESM3_ESM.docx]
