# Supplementary material for: Unilateral or bilateral drainage for patients with bilateral chronic subdural hematoma: a systematic review and retrospective cohort study
Source: Neurosurg Rev. 2025 May 6;48(1):403. doi: 10.1007/s10143-025-03530-0 (PMC12053184; doi:10.1007/s10143-025-03530-0)
Supplement: Supplementary file 4 — Supplementary Material 4 [file 10143_2025_3530_MOESM4_ESM.docx]

**Supplement C. Meta-analyses of non-significant parameters for contralateral treatment**

**Figure 1**

**
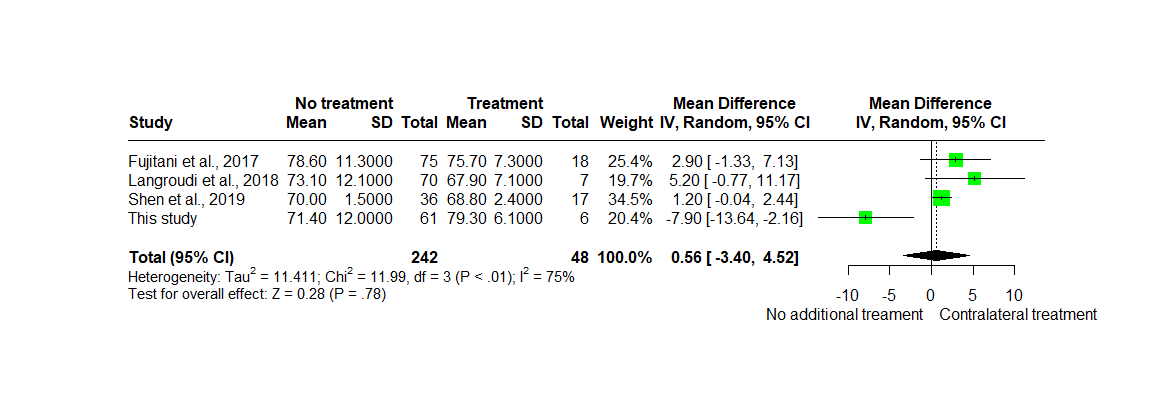
***Forest plot age. Age was not associated with additional contralateral treatment.*

**Figure 2**

**
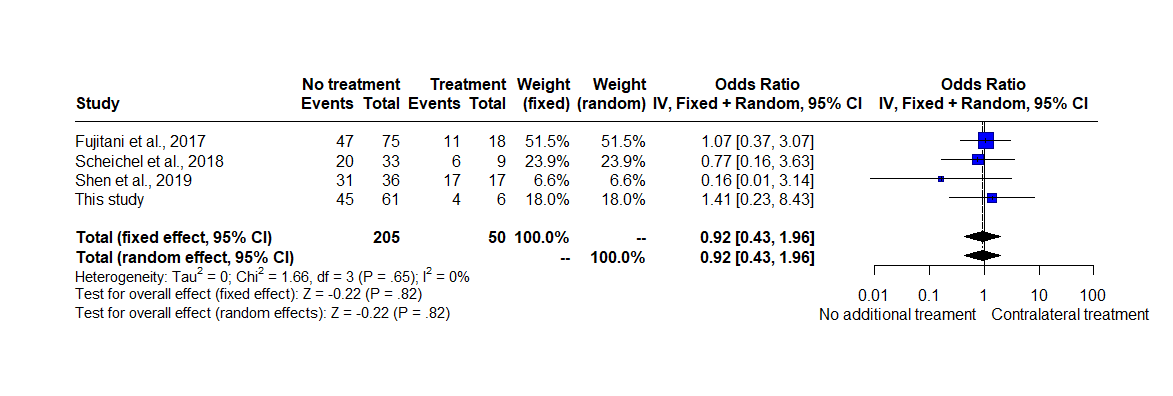
**

*Forest plot male gender. Gender was not associated with additional contralateral treatment.*

**Figure 3**

**
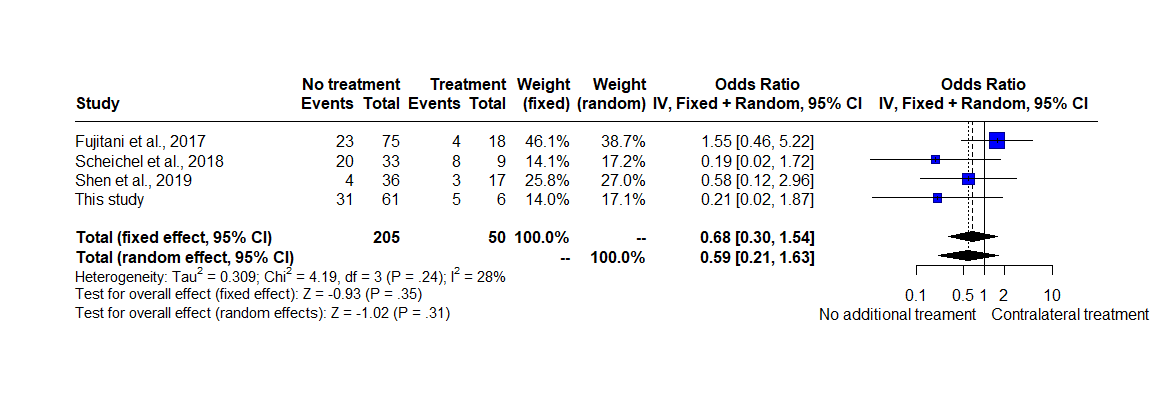
**

*Forest plot anticoagulant and antiplatelet therapy. Use of anticoagulant and antiplatelet therapy was not associated with additional contralateral treatment.*

**Figure 4**

**
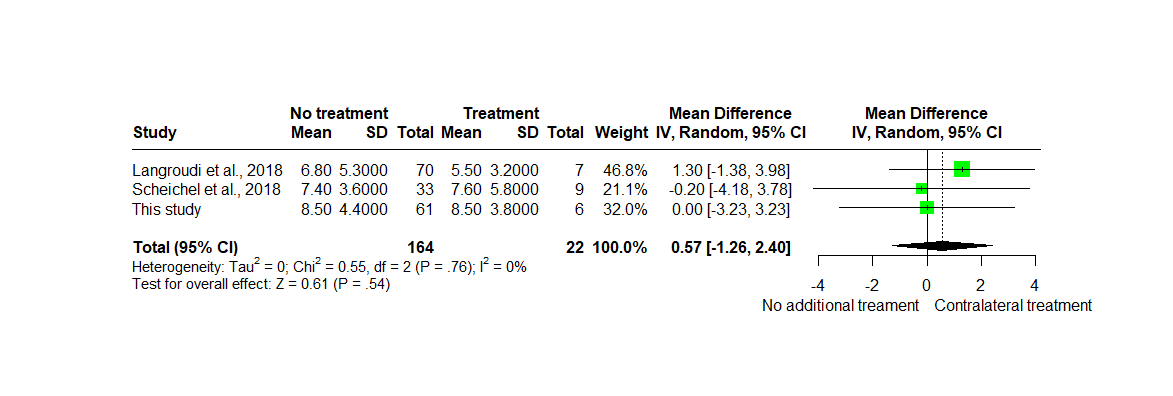
**

*Forest plot midline shift. Amount of midline shift was not associated with additional contralateral treatment.*

**Figure 5**

**
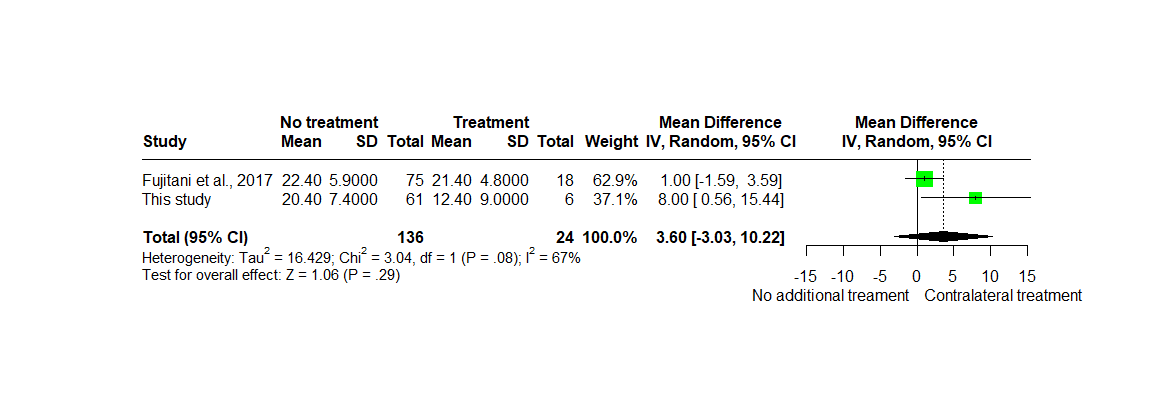
**

*Forest plot ipsilateral hematoma diameter. Maximal ipsilateral hematoma diameter was associated with additional contralateral treatment.*

**Figure 6**

**
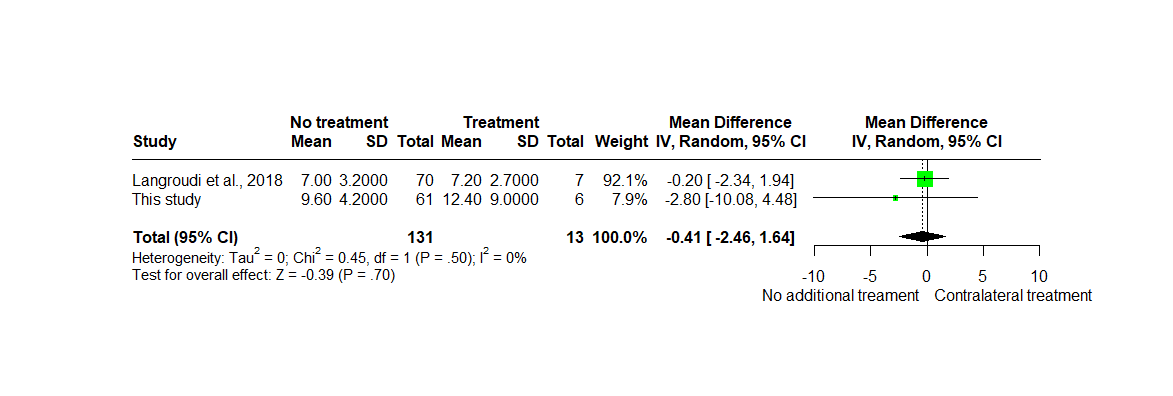
**

*Forest plot contralateral hematoma diameter. Maximal contralateral hematoma diameter was not associated with additional contralateral treatment.*
